# Hetero-Disubstituted Sugarcane Bagasse as an Efficient Bioadsorbent for Cationic Dyes

**DOI:** 10.3390/molecules30153163

**Published:** 2025-07-29

**Authors:** Megg Madonyk Cota Elias Carvalho, Liliane Catone Soares, Oscar Fernando Herrera Adarme, Gabriel Max Dias Ferreira, Ranylson Marcello Leal Savedra, Melissa Fabíola Siqueira, Eduardo Ribeiro de Azevedo, Leandro Vinícius Alves Gurgel

**Affiliations:** 1 Physical Organic Chemistry Group, Department of Chemistry, Institute of Exact and Biological Sciences, Federal University of Ouro Preto, Campus Morro do Cruzeiro, Rua Quatro, 786, Bauxita, Ouro Preto 35402-136, MG, Brazil; megg.carvalho@ufop.edu.br (M.M.C.E.C.); liliane.catone@ufop.edu.br (L.C.S.); 2 Interdisciplinary Research Group on Biotechnology Applied to Agriculture and the Environment, School of Agricultural Engineering, University of Campinas, Avenida Cândido Rondon, 501, Campinas 13083-875, SP, Brazil; oscarf@unicamp.br; 3 Laboratory of Physical Chemistry and Environmental Chemistry, Department of Chemistry, Institute of Exact and Biological Sciences, Federal University of Ouro Preto, Campus Morro do Cruzeiro, Rua Quatro, 786, Bauxita, Ouro Preto 35402-136, MG, Brazil; gabriel.ferreira@ufop.edu.br; 4 Molecular Simulation of Materials Group (MolSMat/LabSimCo), Department of Physics, Institute of Exact and Biological Sciences, Federal University of Ouro Preto, Campus Morro do Cruzeiro, Rua Quatro, 786, Bauxita, Ouro Preto 35402-136, MG, Brazil; ranylson.savedra@gmail.com (R.M.L.S.); melissa@ufop.edu.br (M.F.S.); 5 Department of Physics and Interdisciplinary Science, São Carlos Institute of Physics, University of São Paulo, Av. Trabalhador São-Carlense, 400, São Carlos 13566-590, SP, Brazil; azevedo@ifsc.usp.br

**Keywords:** organic contaminants, agricultural byproduct, water treatment, textile dyes, isothermal titration calorimetry, adsorption mechanism, multicomponent adsorption, density functional theory

## Abstract

A hetero-disubstituted sugarcane bagasse (HDSB) was prepared by simultaneous one-pot chemical modification of sugarcane bagasse with succinic and phthalic anhydrides. HDSB was used in batch mode for the removal of the cationic dyes auramine-O (AO) and safranin-T (ST) from spiked aqueous solutions. Adsorption of the dyes in mono- and bicomponent systems was investigated as a function of HDSB dosage, pH, contact time, and initial dye concentration. Maximum adsorption capacities for AO and ST on HDSB, at pH 7.0, were 1.37 mmol g^−1^ (367.7 mg g^−1^) and 0.93 mmol g^−1^ (293.3 mg g^−1^), respectively. In the bicomponent system, ST was preferentially adsorbed on HDSB, revealing an antagonistic effect of ST on AO adsorption. Changes in the enthalpy of the adsorption as a function of HDSB surface coverage were determined by isothermal titration calorimetry, with Δ_ads_*H*° values for AO and ST equal to −22.1 ± 0.3 kJ mol^−1^ and −23.44 ± 0.01 kJ mol^−1^, respectively. Under standard conditions, the adsorption of the dyes on HDSB was exergonic and enthalpically driven. Desorption removed ~50% of the adsorbed dyes, and subsequent re-adsorption showed that HDSB could be reused, with non-desorbed dye molecules acting as new binding sites. The interaction between AO and ST with HDSB was elucidated by molecular dynamics simulations with atomistic modeling.

## 1. Introduction

The global consumption of dyes and pigments is expected to show an annual increase of 5.3% by 2030, so the disposal of hazardous waste derived from the production and application of these substances will also increase [1]. Textile industries dominate the dyes and pigments market (over 60%), while this sector is highlighted as one of the main industrial activities in terms of water consumption, making it responsible for ~20% of global water pollution [1,2].

Textile industry effluents contain large amounts of organic and inorganic pollutants, including suspended solids, originating from the fixing, bleaching, and dyeing steps of the processes [3]. Consequently, water bodies that receive highly colored textile industry effluents present reduced sunlight penetration, increased biochemical oxygen demand (BOD) and chemical oxygen demand (COD), and altered pH [4]. According to Yaseen and Scholz [5], the typical BOD and COD values of textile effluents are in the ranges of 80–6000 mgO_2_ L^−1^ and 150–30,000 mgO_2_ L^−1^, respectively, while the pH typically ranges from 5.5 to 11.8.

Cationic dyes such as auramine-O (AO) and safranin-T (ST) contribute to this problem, since these dyes are highly stable and bioaccumulative, exerting toxic, carcinogenic, and mutagenic effects on living beings, even at low concentrations [6]. Furthermore, their complex structures provide chemical resistance to heat, light, oxidizing agents, and biodegradation [7]. Therefore, the effective treatment of effluents containing colored dyes is crucial to minimize their environmental impacts.

Several physical, chemical, and biological treatment methods have been developed and applied for dye removal, such as chemical oxidation [8], ozonation [9], ion exchange [10], membrane separation [11,12], and adsorption [13]. However, drawbacks that have been reported include limited applicability, the need for expensive and non-environmentally friendly inputs, and difficulty in recovering and reusing the materials. Furthermore, maximum COD removal of only around 80% has been reported using single biological or physicochemical methods to treat effluents containing dyes [14]. Adsorption employing biomaterials can be highlighted due to its simplicity, versatility, high efficiency, and low operational cost [15,16]. For low concentrations of pollutants, it can be applied as a single treatment method, while it is also promising as a tertiary treatment step, mainly when combined with biological and physicochemical treatments. There are many possible types of biomaterials and chemical modifications that can be used to obtain bioadsorbents that offer high selectivity and efficiency [14].

In this study, a bioadsorbent was produced from sugarcane bagasse (SB), the fibrous residue remaining after the extraction of sugarcane juice in sugar and bioethanol plants in Brazil. According to data provided by the Brazilian Agricultural Research Corporation (Embrapa, a public company linked to the Ministry of Agriculture and Livestock), the amount of SB produced, composed of an average of 46% fiber and 50% moisture, depends on the fiber content of the processed sugarcane, with approximately 250–280 kg of SB generated per ton of sugarcane processed [17]. Therefore, it is one of the most important byproducts for the sugar and alcohol industry. Its main applications are as boiler fuel to produce bioelectricity, a source of hemicelluloses and cellulose to produce biofuels and bioproducts, and feed for confined cattle. However, since Brazil is the world’s largest producer of SB, accounting for 24% of global production, corresponding to 43.7 million tons in 2024/2025 [18], adding value to SB is essential for advancing towards implementation of the principles of a circular bioeconomy.

Although various agricultural residues, including hardwood, softwood, grasses, fruit pits, and vegetable peelings, can be employed as bioadsorbents in either raw or chemically modified forms, grasses such as SB are more readily modified than hardwood and softwood [19]. This enhanced reactivity is primarily attributed to inherent differences in the chemical composition and ultrastructure of the lignocellulosic matrix, particularly in cellulose, hemicelluloses, and lignin. Lignin derived from grasses, which typically contains *p*-hydroxyphenyl (H), guaiacyl (G), and syringyl (S) units, is characterized by greater structural diversity and a lower degree of condensation. This is largely due to a higher proportion of β–O–4 bonds and a lower proportion of C–C bonds, resulting in a more reactive framework for chemical functionalization [19,20]. Additionally, grasses exhibit unique anatomical features as monocotyledons, including cellulose microfibrils with lower degrees of polymerization and crystallinity, as well as hemicelluloses (arabinoxylans) that are more extensively branched and acylated with ferulic and *p*-coumaric acids. These characteristics significantly enhance the accessibility and reactivity of the lignocellulosic matrix to chemical reagents [20]. As a result, bioadsorbents derived from grasses can be extensively functionalized without compromising key physicochemical properties. Notably, they often maintain low swelling capacity, reducing risks of channeling and clogging in fixed-bed column systems, as well as high chemical stability, preventing secondary pollution and preserving adsorption efficiency over multiple reuse cycles due to the minimal degradation of functional groups [21].

A hetero-disubstituted sugarcane bagasse (HDSB) bioadsorbent for the removal of AO and ST was prepared by simultaneous one-pot chemical modification of SB with phthalic and succinic anhydrides. Although phthalic and succinic anhydrides are traditionally produced by the petrochemical industry [22], efforts are being made to develop more sustainable processes for the production of both anhydrides. For example, succinic acid can be produced by bacterial fermentation of carbohydrates [23]. There is also a patent [24] that presents a method for producing succinic acid or its cyclic anhydride form from biomass, under mild conditions and with medium to high yield, without relying on fermentation. The approach involves converting biomass into levulinic acid, which is then transformed into succinic acid by thermal treatment in the presence of nitric acid, optionally using vanadium pentoxide as a catalyst. Recently, Gao et al. [25] reported the production of succinic anhydride from biobased furanic platform compounds, using a visible light-induced oxygenation process.

Mahmoud et al. [26] reported the synthesis of phthalic anhydride from biomass-derived furan and maleic anhydride, employing a two-step process involving a Diels–Alder cycloaddition followed by a dehydration reaction. Shao et al. [27] reported a more sustainable approach for producing phthalic anhydride from bio-derived furan and maleic anhydride. Using a solid acid resin catalyst, the two-step process consisted of a Diels–Alder reaction followed by dehydration, both occurring under relatively mild conditions. According to the authors, the resin catalyst could be reused multiple times, without a significant loss of activity [27].

Pyridine can be recovered by simple or fractional distillation, depending on the complexity of the mixture. However, there are also studies that aimed to synthesize it through more sustainable routes, as reported by Xu et al. [28], Jassem et al. [29], and Kasana et al. [30].

Batch adsorption studies were performed using mono- and bicomponent systems, considering the effects of HDSB dosage, solution pH, contact time, and initial dye concentration. Kinetic and isotherm studies of the adsorption of AO and ST on raw SB were performed to evaluate the feasibility of the proposed chemical modification. Thermodynamic parameters for the adsorption of AO and ST on HDSB were determined to elucidate the mechanism of adsorption. Interactions between AO and ST with HDSB were investigated using infrared spectroscopy measurements of HDSB loaded with AO or ST. Molecular dynamics simulations with atomistic modeling were performed to further understand the interactions of the dyes with HDSB. Dye desorption was also investigated to evaluate the potential reuse of the adsorbent. The ability to perform multiple adsorption–desorption–re-adsorption cycles is essential for advancing the sustainability of the process.

## 2. Results and Discussion

### 2.1. Characterization of the Biomaterials

HDSB presented *wg* = (60 ± 1) %. Although the specific surface area of the material decreased after chemical modification (Supplementary Table S1), the adsorption capacity of HDSB was substantially higher than that of SB (Table 1 and Table 2). This indicated that the adsorption capacity of HDSB was governed by surface diffusion, rather than a pore diffusion mechanism or a combination of both. A detailed discussion of the nature of the adsorbent–adsorbate interaction is provided below in Section 2.2.3.

**Figure 1:**
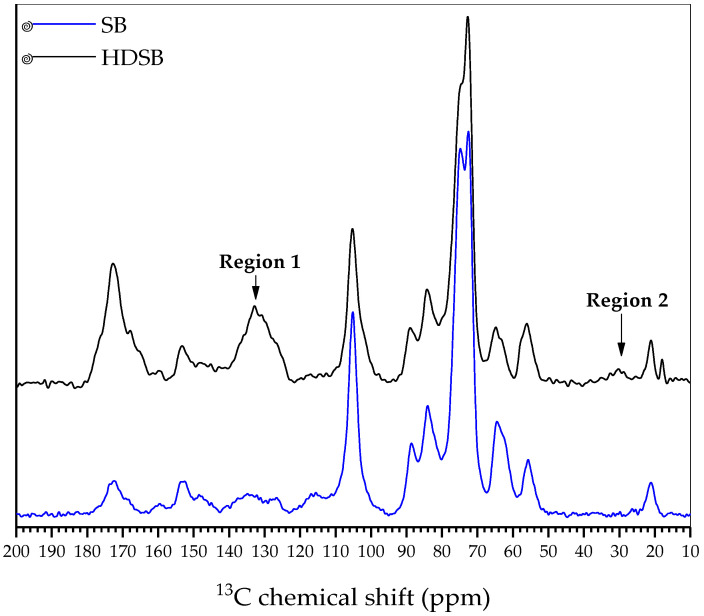
^13^C SS NMR spectra of SB and HDSB.

Figure 1 presents the ^13^C SS NMR spectra of SB and HDSB. For SB, prominent signals in the region 50–120 ppm could mainly be attributed to cellulose, as well as to lignin and hemicelluloses, to a lesser extent [31]. Signals related to the β-D-anhydroglucose units of cellulose were observed at 105 ppm (anomeric carbon C-1), 84–89 ppm (carbon C-4), 72–75 ppm (carbons C-2, C-3, and C-5), and 62 and 65 ppm (carbon C-6) [32]. Chemical shifts observed after chemical modification (denoted “region 1” and “region 2” in the HDSB spectrum) indicated that the modification had been successful. A broad signal centered at 130 ppm (“region 1”) corresponded to the aromatic carbons of the phthalyl units incorporated by the chemical modification, together with signals for the lignin aromatic units, observed at 120–140 ppm in the SB spectrum [33]. The appearance of signals at 24–40 ppm (“region 2”) reflected the presence of methylene carbon atoms of the succinyl units also incorporated after the chemical modification [34]. No substantial changes were observed in the SB signals, suggesting that the main polymeric matrix composed of cellulose, hemicelluloses, and lignin remained unchanged, with no apparent solubilization and loss of the components.

**Figure 2:**
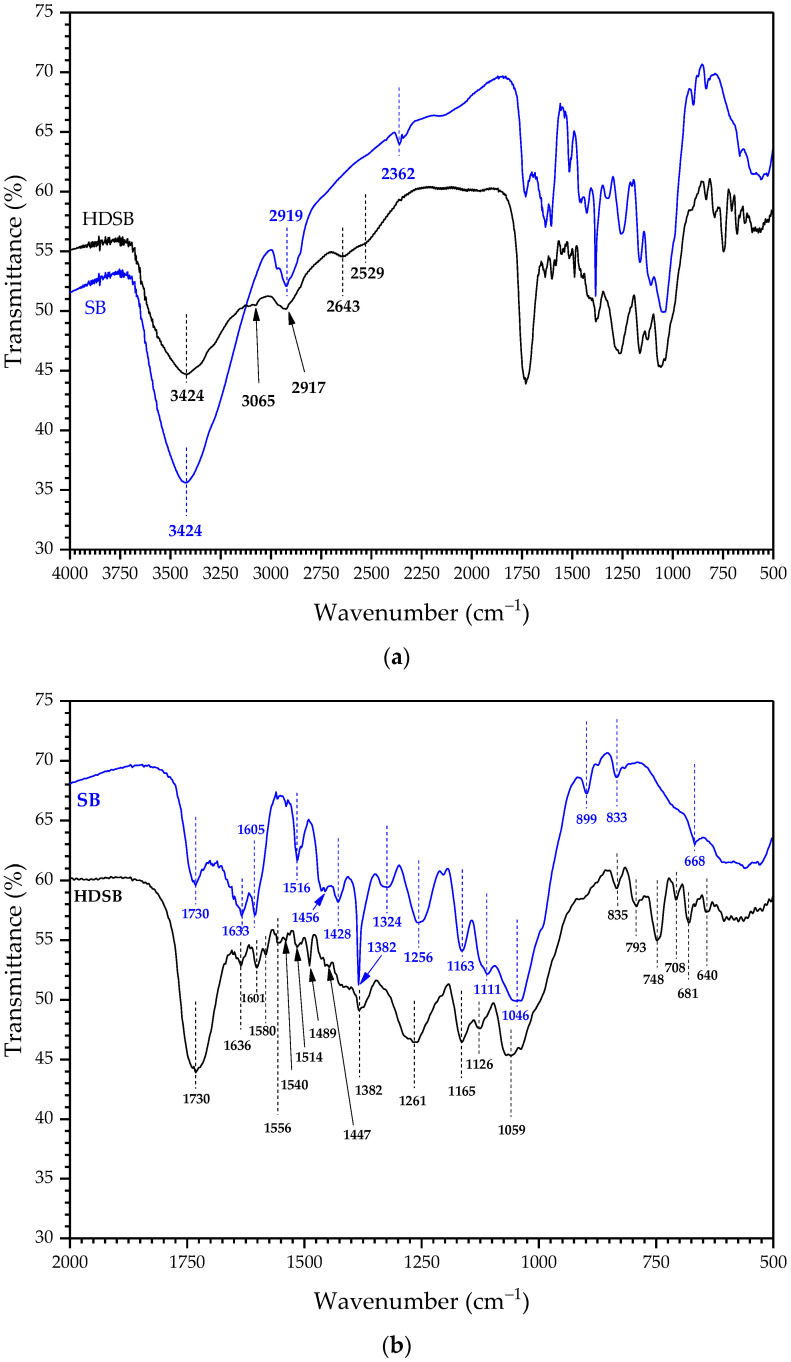

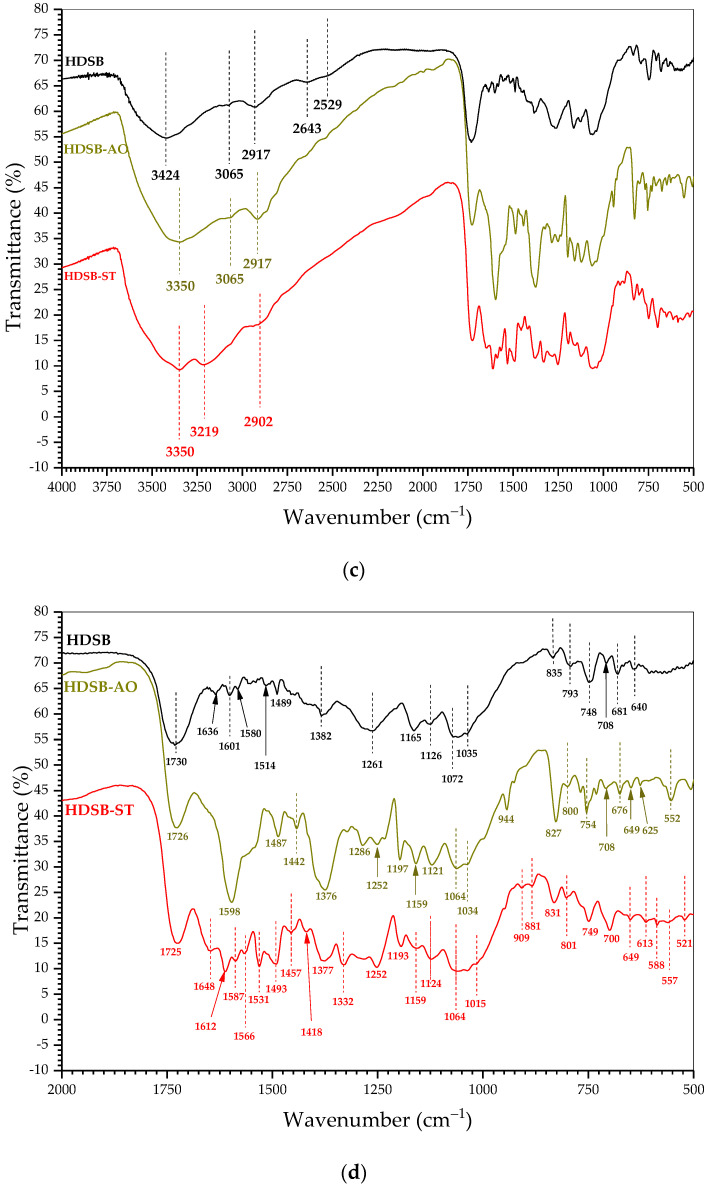
FT-IR spectra of SB and HDSB at (**a**) 4000–500 cm^−1^ and (**b**) 2000–500 cm^−1^, and of HDSB and HDSB loaded with AO (HDSB-AO) and ST (HDSB-ST) at (**c**) 4000–500 cm^−1^ and (**d**) 2000–500 cm^−1^. For comparison purposes, the HDSB-AO spectrum is shifted vertically at +16.008.

The assignment of the FT-IR bands to the respective functional groups was made based on published data [35], together with the other sources cited below. Comparing the FT-IR spectra of SB and HDSB (Figure 2a), the main changes caused by the chemical modification were as follows: (i) appearance of a subtle peak at 3065 cm^−1^, which could be attributed to νC_2sp_^2^–H bonds, indicating the presence of aromatic groups such as those introduced by phthalic anhydride; (ii) decrease in the peak at around 2919 cm^−1^ associated with νC–H of methyl (CH_3_)/methylene (–CH_2_–) groups. This decrease suggested modification of the aliphatic chains of cellulose or hemicelluloses, due to reaction with carboxylic acid anhydrides; (iii) appearance of two subtle peaks at 2643 and 2529 cm^−1^, which could be explained by the formation of carboxylic acid dimers involved in intra- and/or intermolecular hydrogen bonding; (iv) splitting of the peak at 1605 cm^−1^ usually related to aromatic νC=C from lignin and/or phthalyl moieties, or C=O stretching of acids/carbonyls. The splitting could indicate the introduction of new carbonyl groups with different symmetries (such as dicarboxylic acids derived from anhydrides); (v) appearance/intensification of peaks at 1556 and 1540 cm^−1^ suggestive of bending modes of the aromatic ring or conjugated δC=O/C=C vibrations, consistent with the introduction of phthalyl groups; (vi) appearance/intensification of a peak at 1489 cm^−1^ related to νC=C in aromatic rings (from lignin or phthalyl groups), reinforcing the presence of new aromatic groups added by means of phthalyl groups; (vii) disappearance/reduction in the peak at 1428 cm^−1^ related to δC–H and cellulose backbone vibrations. The reduction in this band could indicate modifications in the cellulose backbone due to esterification of hydroxyl groups with carboxylic acid anhydrides; (viii) disappearance/reduction in the peak at 899 cm^−1^ corresponding to a typical twisting vibration of amorphous cellulose, associated with the β-glycosidic bond [36]. This could be related to structural changes or conformational reorganization of cellulose; (ix) appearance of intense peaks at 793 and 748 cm^−1^ related to out-of-plane δC–H in aromatic rings, strongly indicating the introduction of phthalyl units; (x) increase in the peak at 681 cm^−1^ associated with out-of-plane δC–H of substituted aromatic systems (such as phthalyl rings), further corroborating the introduction of aromatic groups; (xi) decrease in the peak at 640 cm^−1^ related to vibrations of groups attached to the lignin backbone, as an indirect effect of the chemical modification. It should be noted that the narrow peak at 1382 cm^−1^ and the peak at 2362 cm^−1^ were related to the presence of atmospheric CO_2_ in the optical path of the spectrometer or adsorbed onto the sample [37,38].

Considering the FT-IR spectra of HDSB loaded with AO (HDSB-AO) and ST (HDSB-ST) (Figure 2c,d), it is important to point out that many of the absorption peaks of the pure dyes overlapped with those of HDSB. As a result, it was challenging to accurately distinguish the spectral changes caused solely by the adsorption phenomenon. For example, in the case of the HDSB-AO spectrum, the peaks at 1598, 1376, 1159, 1064, 944, 754, and 676 cm^−1^, among others, could be related to AO [39]. In the case of the HDSB-ST spectrum, the peaks at 1612, 1531, 1493, 1418, 1332, 1252, 1193, 1015, 881, 831, 801, 700, 613, and 588 cm^−1^, among others, could be associated with ST [40]. Nevertheless, comparing the FT-IR spectra of HDSB-AO and HDSB-ST with the HDSB spectrum, changes that could be highlighted were: (i) broadening of the band in the 3400–3300 cm^−1^ region, suggesting involvement of the R–OH groups in dye adsorption, and (ii) a shift in the band at 1730 cm^−1^ (related to νC=O in R–COOH) to 1725–1726 cm^−1^, for HDSB loaded with AO and ST, attributed to interaction of C=O with the dyes. The HDSB-AO spectrum showed the following changes that could be related to the adsorption of AO: (i) disappearance of the peaks at 1636 and 1580–1514 cm^−1^, and (ii) appearance of peaks at (or shifting to) 1286, 1197, 1034, 827, 800, and 649 cm^−1^. In the HDSB-ST spectrum, the following changes could be related to the adsorption of ST: (i) disappearance of the peaks at 2643, 2529, and 1514 cm^−1^, and (ii) appearance of peaks at (or shifting to) 2902, 1587, 1566, 1377, 1252, 1159, 1064, 909, 749, 649, 557, and 521 cm^−1^.

The peaks at 2643 and 2529 cm^−1^ were characteristic of hydrogen bonding in carboxylic acid dimers (which is common after the introduction of free R–COOH groups by esterification reaction with anhydrides), so their disappearance suggested that these groups were involved in the adsorption of AO and ST. The wavenumber range 1650–1540 cm^−1^ is related to asymmetric νCO_2_^−^ (carboxylate ion), while 1440–1395 cm^−1^ is related to in-plane δO–H of carboxylic acid, 1450–1360 cm^−1^ is related to symmetric νCO_2_^−^, 1320–1210 cm^−1^ is related to νC–O of carboxylic acid, 1270–1010 cm^−1^ is related to asymmetric νC–O–C of ether, 1060–1035 cm^−1^ is related to νC–O of non-cyclic acid anhydrides, and 960–900 cm^−1^ is related to out-of-plane δO-H of carboxylic acids [41]. Therefore, the changes observed in the FT-IR spectra of HDSB-AO and HDSB-ST suggested that carboxylate groups from phthalyl and succinyl moieties were involved in adsorption of the dye molecules. The changes below 990 cm^−1^ suggested that adsorption of AO and ST affected the vibration modes of C–H bonds from the lignocellulosic backbone or from the dyes themselves [42,43].

The surfaces of HDSB-AO and HDSB-ST, after monocomponent adsorption, and of HDSB-AO-ST, after bicomponent adsorption, were mapped using SEM-EDX spectroscopy. In general, the radial distribution function (RDF) results showed that the two dyes exhibited similar behavior in their interactions with the HDSB surface. However, a notable interaction between AO and the phthalyl group of HDSB was observed, indicative of high affinity and structured arrangements. In the case of ST, besides its affinity for the HDSB surface, the ST molecules also interacted with each other, as indicated by the intense imine peak, as will be shown in Section 2.5.

### 2.2. Batch Adsorption Studies

#### 2.2.1. Effect of Adsorbent Dosage

Supplementary Figure S2 shows plots of *q*_e_ and *R* for AO and ST, as a function of HDSB dosage. The decreasing behavior of the *q*_e_ curves for AO and ST, as a function of HDSB dosage, could be attributed to the greater quantity of available surface active sites at higher HSDB dosages, leading to lower adsorbate/adsorbent ratios, implying a higher driving force for dye adsorption and resulting in higher *R* values, since the initial dye concentration was constant for all the HDSB dosages used. However, this also resulted in lower capacity to accommodate dye species on the surface of HDSB, considering repulsive and electronic effects inherent to a surface overpopulated with dye molecules, as observed at lower HDSB dosages. Despite this, higher HDSB dosages resulted in increased removal efficiencies for both dyes. Similar results were reported elsewhere for the adsorption of different dyes on raw and chemically modified sugarcane bagasse [44,45,46]. For both dyes, a dosage of 0.2 g L^−1^ was selected in the subsequent studies, since it combined a high adsorption capacity with a higher removal percentage than obtained using a dosage of 0.1 g L^−1^. Furthermore, the dosage of 0.2 g L^−1^ (or 20.0 mg of HDSB in 100.0 mL of dye solution) was more suitable because the use in adsorption studies of very low dosages of heterogeneous materials with larger particle size, such as HDSB (32 mesh, 0.5 mm), can lead to high standard deviations, due to non-uniform functionalization of the surface.

#### 2.2.2. Effect of Solution pH

**Figure 3:**
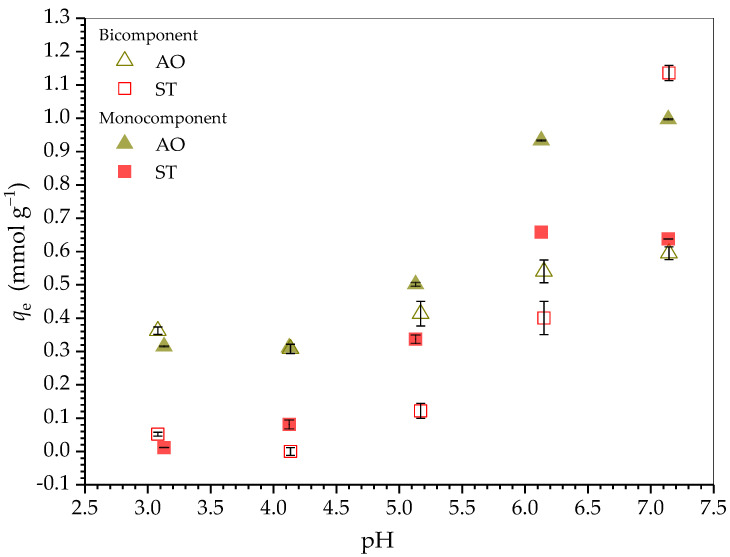
Effect of solution pH on the mono- and bicomponent adsorption of AO and ST on HDSB (0.374 mmol L^−1^ AO and/or ST, 25.0 ± 0.1 °C, 130 rpm, 24 h, and 0.2 g L^−1^ HDSB).

Figure 3 shows the effect of solution pH in the range 3.1–7.2 on the equilibrium adsorption capacity (*q*_e_) for AO and ST on HDSB. At pH values above the pH_PZC_ of HDSB (2.69 ± 0.04, Supplementary Figure S3a), adsorption of the cationic dyes was favored by the negative net surface charge of HDSB. Therefore, for both mono- and bicomponent systems, the *q*_e_ values increased with increasing solution pH, suggesting the occurrence of adsorbent-adsorbate electrostatic interactions.

Up to pH 7.0, 100% of the AO species were protonated (Supplementary Figure S4a), reflecting the increase in the *q*_e_ values with increasing pH, which was corroborated by the increased negative charge density of the adsorbent with increasing pH (Supplementary Figure S3b). Therefore, the adsorption of AO appeared to preferentially occur by electrostatic interaction. On the other hand, ST was positively charged throughout the pH range studied, due to its pH-independent positive charge on the quaternary nitrogen atom of the imine group. Both positively charged dyes could form strong interactions with the negatively charged HDSB surface, but the condensed rings of ST could also interact more efficiently by π interactions with the phthalyl groups on the HDSB surface, compared to AO. Studies involving analogous systems (such as indole-benzene) have demonstrated that heteroaromatic compounds, such as ST, can form π–π stacking interactions with benzene rings [47,48]. Therefore, ST adsorption could have occurred by interactions other than the electrostatic mechanism, such as π-stacking [49]. This hypothesis was reasonable, since ST adsorption in a monocomponent system was negligible below pH 4.0, although 100% of the ST species were in the form of ST^2+^ and the HDSB surface was negatively charged at this pH. Furthermore, despite the substantial increase of *q*_e_ from pH 5.0 onwards, at which around 96% of the ST species were in the form of ST^2+^, the *q*_e_ value remained constant at 6.0 < pH < 7.0, at which about 80% of the ST species were in the form of ST^+^. This suggested that electrostatic interactions were not predominant, and that ST^+^ was adsorbed preferentially to ST^2+^. In addition, considering the results and the chemical properties of the dyes, the greater hydrophobicity of ST was likely to play an important role in its adsorption. Since this dye was positively charged, water should act as a dielectric on the quaternary nitrogen atom of the imine group.

For the bicomponent system, in most of the pH range studied, the *q*_e_ values for both dyes were lower than those obtained for the monocomponent systems, indicating an antagonistic interaction between AO and ST. This difference became larger above pH 5.0 (analyzing each dye individually), especially for AO, which presented substantially lower *q*_e_ values in the presence of ST (about 30% at pH 7.14), while the *q*_e_ values for ST were less affected by the presence of AO species. Consequently, in the bicomponent system, the *q*_e_ values for ST exceeded the *q*_e_ values for AO at pH 7.14. Since the *q*_e_ values for ST were less affected by the presence of AO species, this suggested that ST was preferentially adsorbed at specific adsorption sites. Based on the results (discussed in Section 2.5), both dyes preferentially interacted with the phthalyl group. However, ST also tended to become structured at short distances around the succinic group, which was not observed for AO.

Therefore, considering the results for the adsorption of AO and ST on HDSB in mono- and bicomponent systems, the kinetics and equilibrium studies were performed at pH 7.0.

#### 2.2.3. Adsorption Kinetics

**Figure 4:**
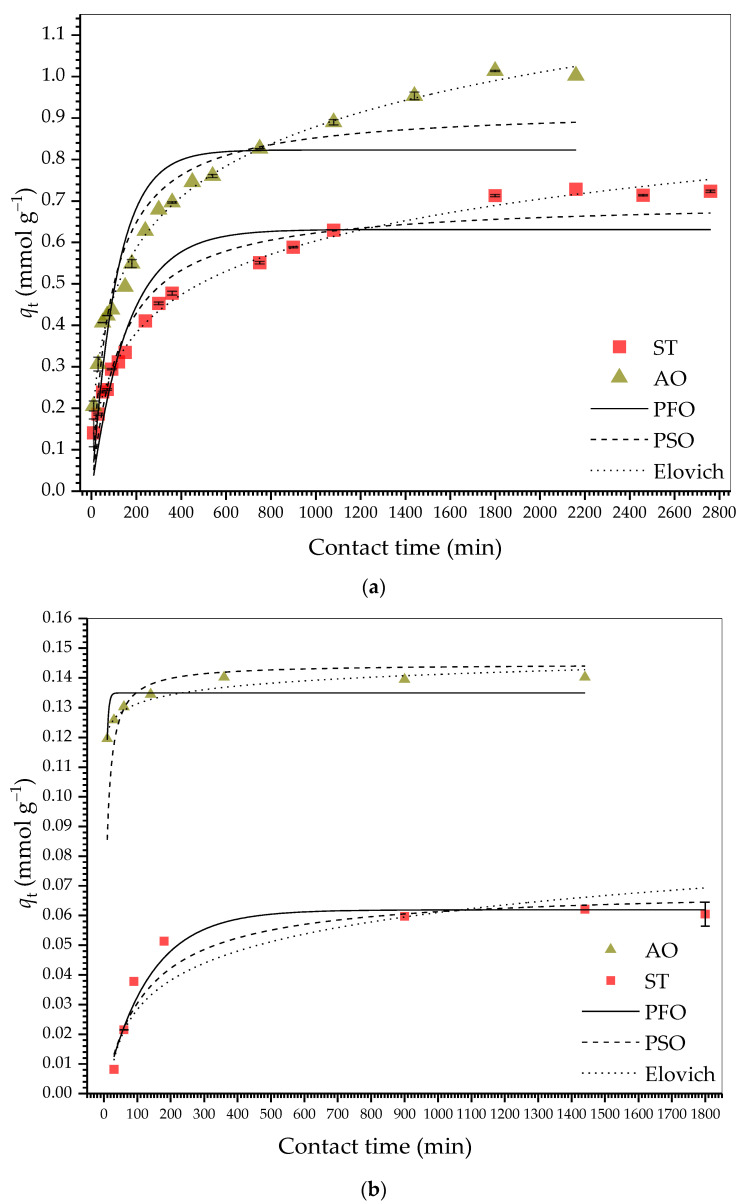

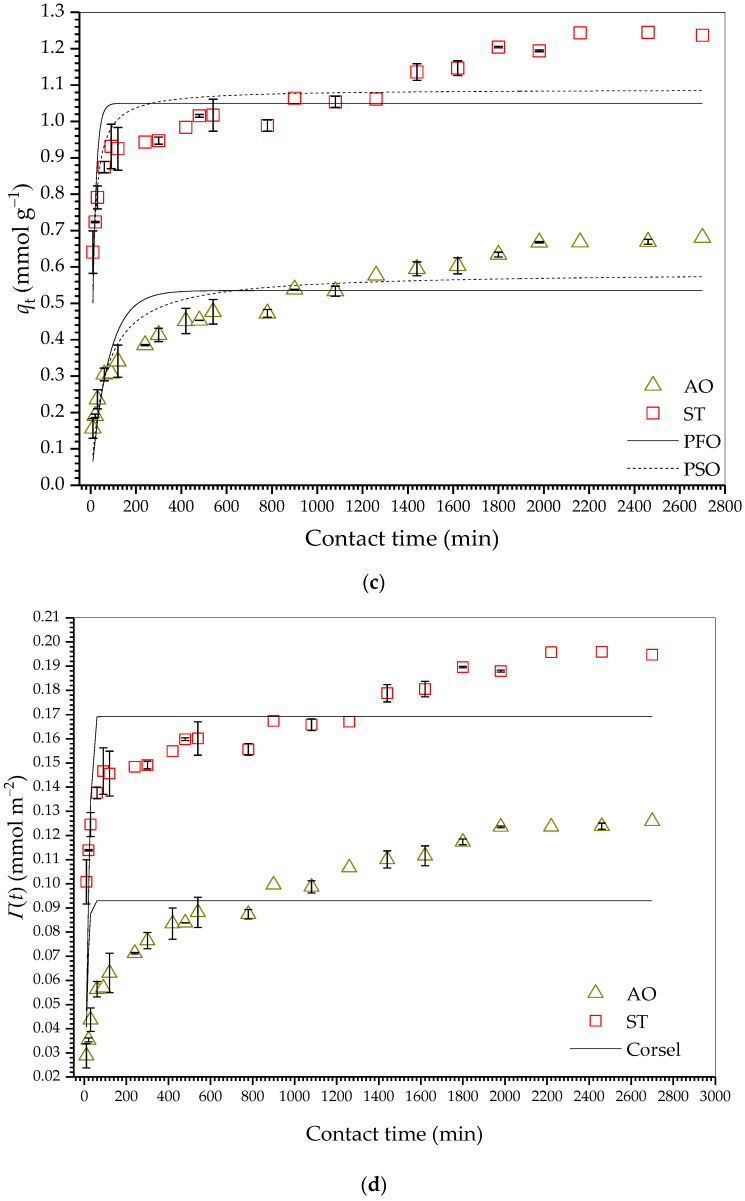
Monocomponent adsorption kinetics of AO and ST on (**a**) HDSB and (**b**) SB, with the experimental curves fitted using the PFO, PSO, and Elovich models. Bicomponent adsorption kinetics of AO and ST on HDSB, with the experimental curves fitted using (**c**) the PFO and PSO models, and (**d**) the Corsel model (*C*_AO_ = *C*_ST_ = 0.374 mmol L^−1^, 0.2 g L^−1^ adsorbent, 130 rpm, 25.0 ± 0.1 °C, and pH 7.0).

Figure 4a–d shows the kinetic curves for adsorption of AO and ST on HDSB and SB. Table 1 provides the kinetic parameters estimated from modeling the experimental data for the monocomponent adsorption systems, applying the PFO [50], PSO [51], Elovich [52], IPD [53], and Boyd [54] models.

For the monocomponent systems, *q*_t_ for both dyes increased substantially up to 300 min, reaching equilibrium at 1800 and 2460 min for the adsorption of AO and ST, respectively, on HDSB (Figure 4a), and at 360 min and 900 min for the adsorption of AO and ST, respectively, on SB (Figure 4b). The faster adsorption of AO on both SB and HDSB, as well as the shorter equilibrium times for SB, suggested the occurrence of different adsorption mechanisms. Similar behavior was reported by Teodoro et al. [43] and Fideles et al. [42], who investigated the adsorption kinetics of AO and ST on cellulose and sugarcane bagasse modified with trimellitic anhydride, respectively.

For adsorption of both dyes on HDSB, the best fits were obtained with the Elovich model (higher *R*^2^ and *R*^2^_adj_ values, and lower χ^2^_red_ values), indicating the influence of heterogeneity of the HDSB surface on adsorption. Although this model does not allow accurate inferences to be made regarding the main mechanisms involved in the interactions between adsorbate and adsorbent, it provides information about the nature of the adsorption and is highly suitable for evaluation of kinetics involving heterogeneous surfaces [52]. Therefore, it was reasonable to assume the existence of different types of adsorption sites, indicating the possible occurrence of heterogeneous adsorption on the surfaces of the adsorbents (SB and HDSB), which resulted in different adsorption mechanisms. In addition, the *β* (Elovich desorption rate constant) values were much higher than the *α* (Elovich initial adsorption rate constant) values, which could provide an explanation for the longer equilibrium times observed for all the monocomponent systems, with the desorption rate being higher than the adsorption rate, consequently delaying attainment of equilibrium. On the other hand, the 2-fold higher *α* value estimated for the adsorption of ST on HDSB suggested that ST had higher affinity than AO for the HDSB adsorption sites. The presence of the three planar rings in the ST structure could favor stronger interaction with the HDSB surface, compared to AO, where the rings deviate slightly from the plane, as shown in the fully optimized structures obtained for ST and AO by Teodoro et al. [43].

Supplementary Figures S5 and S6 show the IPD (intraparticle diffusion) plots for adsorption of AO and ST on HDSB and SB, respectively. The plots revealed two (SB) and three (HDSB) linear portions, characterized by different slopes. The first linear portion did not cross the origin (*C* ≠ 0), indicating that film diffusion was the step limiting the adsorption rate [53]. The intraparticle diffusion coefficients (*k*_d,i_, with *i* = 1, 2, and 3; Table 1) suggested strong initial attraction between the dyes and the surface active sites of the bioadsorbents.

The Boyd plots for adsorption of AO and ST on HDSB (Supplementary Figure S7) and SB (Supplementary Figure S8) presented initial linear portions and did not intersect the origin, confirming that film diffusion could be considered the step limiting the adsorption rate, as also suggested by the IPD model. For both AO and ST, the effective diffusion coefficients (*D_i_*), calculated from the slopes of the initial linear portions of the plots (*B*), were higher for adsorption on SB than on HDSB. This was because the chemical modifications made the surface of HDSB more hydrophilic (and more solvatable), consequently hindering diffusion of the dyes. The *D_i_* values for HDSB and SB were in agreement with *D_i_* values reported in the literature for adsorption of AO and ST (conditions: pH 7.0, 25 °C, 130 rpm, adsorbent dose of 0.2 g L^−1^) on cellulose (2.14 × 10^−11^ m^2^ min^−1^ for AO and 1.82 × 10^−12^ m^2^ min^−1^ for ST) [43] and sugarcane bagasse (8.39 × 10^−12^ m^2^ min^−1^ for AO and 2.39 × 10^−12^ m^2^ min^−1^ for ST) [42] modified with trimellitic anhydride.

Table 2 presents the kinetic parameters for bicomponent AO-ST adsorption on HDSB, estimated by modeling the experimental data using the PFO [50], PSO [51], and Corsel [55] models. Figure 4c shows plots of *q*_t_ vs. *t*, fitted with the PFO and PSO models. Figure 4d shows plots of *Γ* vs. *t*, fitted with the Corsel model. Although the PFO and PSO models do not consider competitive adsorption [50,51], they can be used as mathematical descriptors to individually describe the adsorption of dye species in solution, by comparing the estimated adsorption rates and the equilibrium adsorption capacities, making them applicable to a wide variety of kinetic datasets [56]. Hence, for both kinetic models, the quality of the fits to the experimental data were also evaluated for bicomponent adsorption.

The adsorption of AO on HDSB reached equilibrium earlier than the adsorption of ST (Table 1), as also observed for the monocomponent adsorptions of AO and ST on HDSB. However, for AO, *t*_e_ increased from 1800 to 1980 min in the bicomponent system, while for ST, *t*_e_ decreased from 2460 to 2160 min (Table 2). This was suggestive of competition between AO and ST for some common adsorption sites. Therefore, two hypotheses could be considered: (i) the occurrence of mobile adsorption, where thermodynamic effects could lead to one dye molecule replacing another dye molecule, although this was not supported by the shape of the adsorption isotherms (Section 2.2.4) [43]; and (ii) a change in the way that a dye molecule approached the surface of HDSB to be adsorbed, as the surface became overpopulated with different dye molecules, related to electronic and steric effects in the bicomponent system, compared to the monocomponent systems, resulting in delayed establishment of equilibrium.

The results shown in Figure 4c also revealed that for the equimolar bicomponent adsorption at pH 7.0, ST was preferentially adsorbed, with 44% higher *q*_e,exp_ for ST, in comparison to the *q*_e,exp_ value obtained for monocomponent ST adsorption (*q*_ST,bi_/*q_S_*_T,mono_ = 1.44), while AO removal was reduced by 33% (*q*_AO,bi_/*q*_AO,mono_ = 0.67). Despite the evident competition between the dyes, which resulted in an antagonistic effect on AO adsorption, the total bicomponent adsorption capacity (*q*_bi,total_ = *q*_AO,bi_ + *q*_ST,bi_) was higher than the individual adsorption capacities (*q*_total,bi_ > *q*_AO,mono_ and *q*_total,bi_ > *q*_ST,mono_). These results reinforced the hypothesis that the adsorption of AO had a greater influence of specific binding sites (phthalyl groups, involving electrostatic attraction), where the interaction with these sites was more susceptible to disruption in the presence of ST^+^ and ST^2+^ species, with apparent greater affinity for ST at pH 7.0. The observations also supported the notion that interaction of the binding sites with ST was less susceptible to interference by other dye species (such as AO^+^) in solution. This was contrary to the findings of Teodoro et al. [43], who reported that the esterification of a cellulose-based adsorbent with trimellitic anhydride resulted in suppression of the adsorption of both AO and ST in a bicomponent system. It is plausible that such different behaviors resulted from the different chemical modifications employed in the studies, corroborating the hypothesis that distinct chemical modifications can enhance the selectivity of adsorbents towards certain pollutants, which is of considerable technological interest.

In the present study, the bi-functionalization of HDSB conferred multifunctional characteristics. The aliphatic chain of the succinic unit presented free rotation around the sigma bond, allowing different adsorption configurations and the creation of electrostatic interaction between carboxylate groups and the cationic dye molecules. At the same time, although the rigid aromatic ring of the phthalyl unit did not allow different adsorption configurations, it enabled the formation of electrostatic interactions between carboxylate groups and the cationic dye molecules, in addition to π-π stacking interactions between the aromatic rings of phthalyl units and the dye molecules. On the other hand, the carboxylic functions of the trimellityl units introduced in the cellulose matrix by Teodoro et al. [43] (mono-functionalization, rather than bi-functionalization) appeared to have resulted in an adsorbent with a more homogeneous surface and a tendency to remove positively charged species by electrostatic and π-π stacking interactions, but with fewer adsorption configurations, compared to HDSB. As a result, there may have been a high degree of competition for the binding sites, which suppressed adsorption in the bicomponent system.

For bicomponent adsorption, the *q*_e_ values estimated by the PSO and PFO models were statistically equal, for both dyes (95% confidence level), and were close to the *q*_e,exp_ values (Table 2). Nevertheless, the PSO model provided the best description of the experimental data for the bicomponent system, since it presented higher *R*^2^ and lower *χ*^2^_red_. The Corsel model [55], which is a competitive kinetic model, provided lower *R*^2^ values, but the fitting parameters could enable better understanding of the bicomponent adsorption system studied. The association constant (*K = k*_+1_/*k*_−1_), which is the ratio between the initial intrinsic adsorption (*k*_+1_) and desorption (*k*_−1_) rate constants, can be used to infer the affinity of the binding sites for a solute “*i*” in the presence of a solute “*j*” [57]. The 10-fold higher *K* value estimated for ST adsorption confirmed the greater affinity of the HDSB binding sites for ST, compared to AO, in the bicomponent system studied.

The adsorption and desorption interaction constants (*α* and *β*) are related to the possible occurrence of lateral interactions between adsorbed species in bicomponent systems [55,57]. The positive *α* values (Table 2) indicated that for both dyes, adsorption was hindered near dye molecules that had already been adsorbed, resulting in a decrease in the adsorption rate function, $k{\left(\Gamma \right)}_{on}^{app}$, as the HDSB surface became saturated [57], with the decrease being greater for ST than AO, mainly due to the higher *α* value for ST adsorption. A possible explanation was the existence of a repulsive potential around each adsorbed dye molecule, which hindered the subsequent attachment of dye species close to those that had already been adsorbed [58]. In addition, steric hindrance caused by adsorbed dye molecules could have restricted the adsorption of new dye molecules on adjacent sites. The higher the *β* value, the higher the increase in the desorption rate function, $k{\left(\Gamma \right)}_{off}^{app}$, as the HDSB surface became saturated [57]. The higher *β* value for ST (Table 2) indicated a greater increase in the desorption rate function as the HDSB surface became saturated, compared to AO. Therefore, it could be concluded that when the HDSB surface approached saturation, the ST adsorption rate decreased faster than the AO adsorption rate, while the ST desorption rate increased slightly faster than the AO desorption rate. Hence, the ST adsorption rate was more negatively affected by the repulsive forces exerted by the dye molecules that had already been adsorbed, compared to AO adsorption, although the *K* value for ST adsorption was much higher than for AO adsorption [58]. These observations provide support for the hypothesis that electrostatic and π interactions were the main mechanisms responsible for the adsorption of AO and ST, respectively.

Additionally, the greater the sum of the interaction constants (*γ* = *α* + *β*), the greater is the influence of a solute “*i*” on the adsorption of a solute “*j*” and, consequently, on the total adsorption capacity [57]. Therefore, for the experimental conditions investigated, ST (with higher *K* and *γ* values) had a greater influence than AO on the total adsorption capacity of HDSB, in agreement with the experimental results for the bicomponent system. Another possible analysis considered the difference between the interaction constants for the two adsorbed dye species (*γ_i_* − *γ_j_*), which indicated the occurrence of some chemical or physical (attractive or repulsive) forces in the bicomponent system that could influence the distribution of the adsorbed dye species on the HDSB surface [59]. As shown in Table 2, the *γ* value was less than zero, indicating that the adsorption of a given species “*i*” was favored over the adsorption of a species “*j*”, resulting in worsening of the removal of the species with lower affinity (in this case, AO).

#### 2.2.4. Adsorption Isotherms

**Figure 5:**
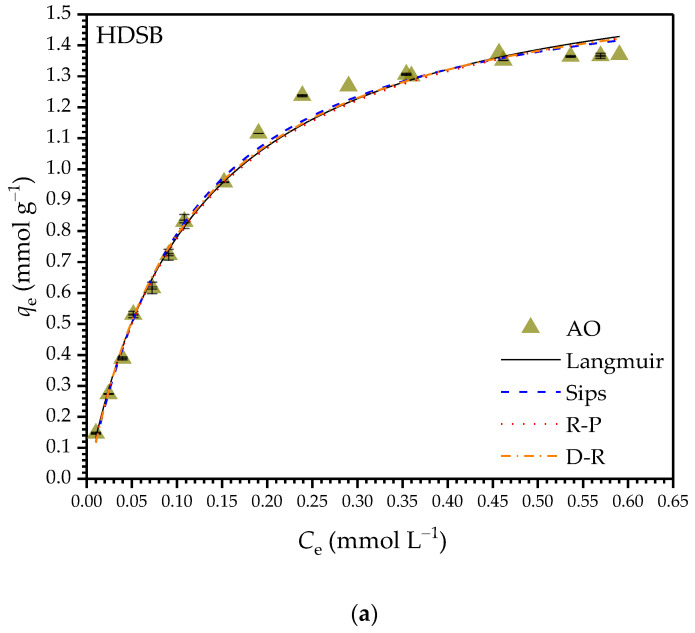

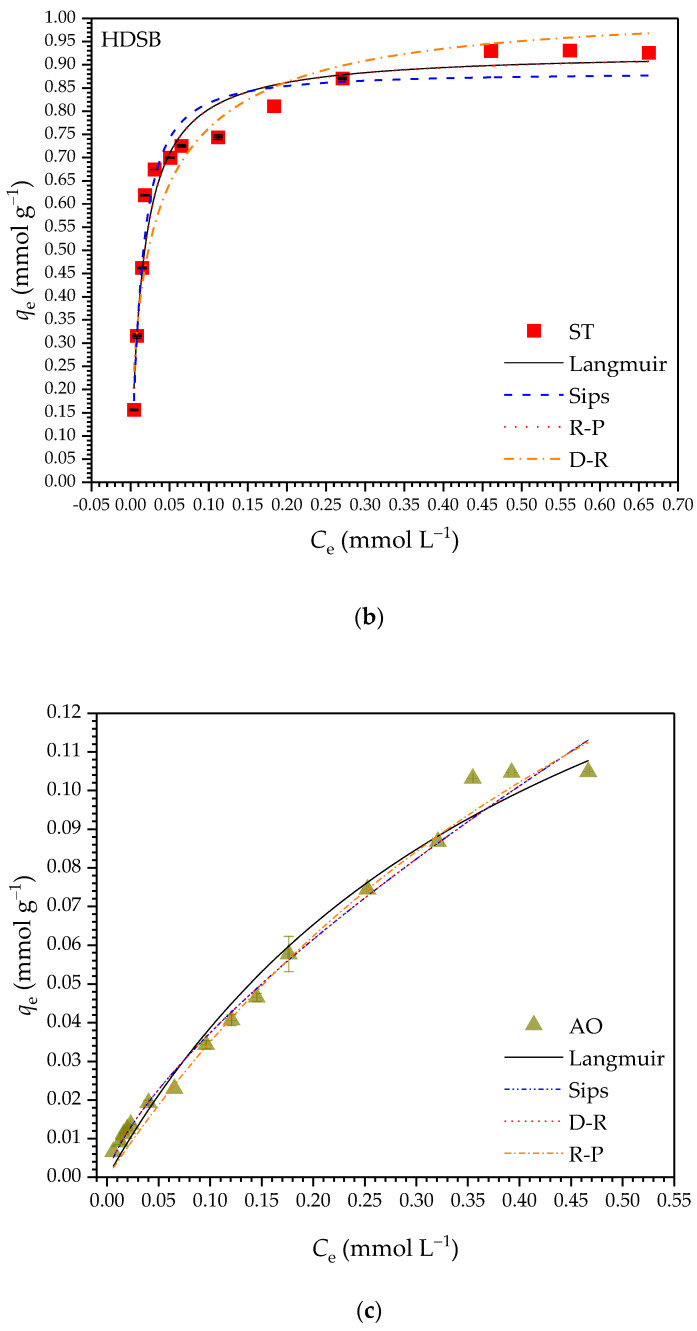

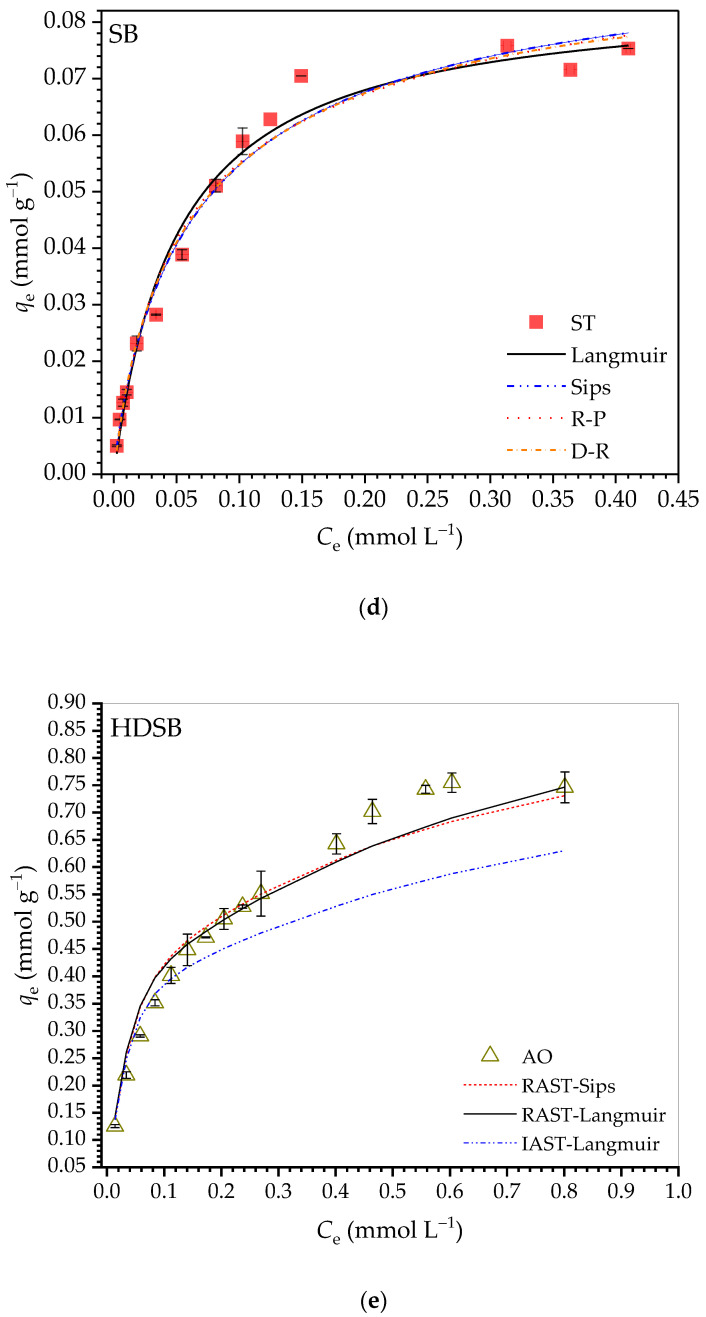

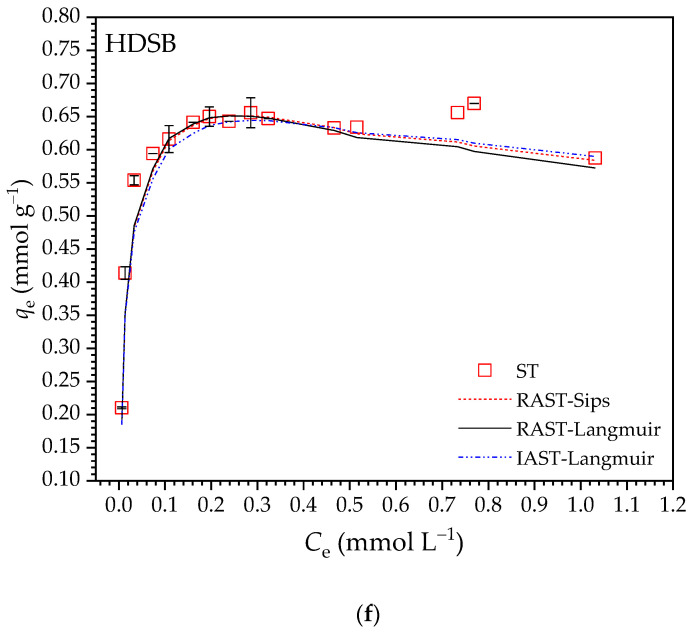
Isotherms for monocomponent adsorption of (**a**) AO and (**b**) ST on HDSB, monocomponent adsorption of (**c**) AO and (**d**) ST on SB, and bicomponent adsorption of (**e**) AO and (**f**) ST on HDSB (0.2 g L^−1^ HDSB or SB, *C_i_*_,AO_ = 0.04–0.94 mmol L^−1^, *C_i_*_,ST_ = 0.05–1.14 mmol L^−1^, pH 7.0, 130 rpm, 25.0 ± 0.1 °C, and *t*_e,AO_ = *t*_e,ST_ = 2280 min).

Figure 5a–d show the isotherms for monocomponent adsorption of AO and ST on HDSB and SB, with the curves fitted using the Langmuir [60], Sips [61], R-P [62], and D-R [63] models. Figure 5e,f show the bicomponent adsorption isotherms, with the curves fitted using the IAST-Langmuir, RAST-Sips [64], and RAST-Langmuir [64] models, which were fed with fitting parameters (*Q*_max,est_, *b*, and *n*) estimated by modeling the monocomponent AO and ST experimental data with the Sips and Langmuir models. Table 3 and Table 4 present the fitting parameters estimated by nonlinear regression analysis of the equilibrium data for the mono- and bicomponent systems, respectively.

The *Q*_max_,_exp_ values for monocomponent adsorption of AO and ST on HDSB were around 10-fold higher than for adsorption on SB. Therefore, the chemical modification improved the efficiency of the bioadsorbent for the removal of AO and ST from spiked aqueous solutions. The shapes of the isotherms (Figure 5a–d) suggested that ST had higher affinity than AO for both bioadsorbents, as well as that both dyes had higher affinity for HDSB than SB.

For mono- and bicomponent adsorption of AO and ST on HDSB, the *q*_e_ values greatly increased at the initial *C*_e_ values, especially for ST adsorption (“box” type isotherm), showing the higher affinity of HDSB for ST than AO. All the models provided good fits to the AO and ST adsorption data, with high *R*^2^_adj_ values and low *χ*^2^_red_ and *RSS* values. For monocomponent adsorption of AO on HDSB, the models overestimated *Q*_max_ by 18–26%. For monocomponent adsorption of ST on HDSB, the Langmuir model accurately predicted *Q*_max_, the Sips and R-P models underestimated *Q*_max_ by 3–5%, and the D-R model overestimated *Q*_max_ by 9%.

The Sips model is a hybrid model that can be reduced to the Langmuir model when the *n* value is close to unity. The R-P model is also a hybrid model, where the *β* values close to unity indicated that the Langmuir model could satisfactorily describe the adsorption behavior. These features suggested that the adsorption of both AO and ST occurred at homogeneous sites (i.e., preferably on phthalyl groups) (see Section 2.5). However, the surface of HDSB presented different adsorption sites (succinyl and phthalyl units) created by the bi-functionalization, as discussed in Section 2.1 and Section 2.3, in addition to the chemical functionalities present in cellulose (unmodified hydroxyl groups), hemicelluloses (hydroxyl groups and ester and carboxylic acid carbonyl groups), and lignin (aliphatic and aromatic hydroxyls, and ketone and carboxylic acid carbonyls). Therefore, the Langmuir model should only be used as a mathematical descriptor of the adsorption behaviors of AO and ST on HDSB.

For bicomponent adsorption of AO-ST on HDSB (Figure 5e,f), at low *C*_e_ values, the *q*_e_ values were higher for ST than AO, as also shown in the kinetic data points corresponding to the equilibrium concentrations (*C*_e,AO_ = 0.37 mmol L^−1^ and *C*_e,ST_ = 0.36 mmol L^−1^). However, when thermodynamic equilibrium was reached, the *q*_e_ values for AO and ST (*q*_e,exp,AO_ = 0.748 ± 0.006 mmol g^−1^ and *q*_e,exp,ST_ = 0.75 ± 0.03 mmol g^−1^) were statistically the same (*t*-test at 95% confidence), confirming that thermodynamic effects also influenced the bicomponent adsorption of AO and ST on HDSB. These values were lower than the *q*_e_ values for monocomponent adsorption of AO and ST on HDSB (*q*_e,exp,AO_ = 1.362 ± 0.009 mmol g^−1^ and *q*_e,exp,ST_ = 0.929 ± 0.003 mmol g^−1^), where the decrease of *q*_e,AO_ was greater than that of *q*_e,ST_, as shown previously (Figure 5a,b,e,f). This behavior was also observed by Teodoro et al. [43], who used cellulose modified with trimellitic anhydride as a bioadsorbent for AO and ST in both mono- and bicomponent systems. However, in the present study, the total adsorption capacity in the bicomponent system was higher than the individual adsorption capacities for each dye in the monocomponent systems. Therefore, HDSB was also shown to be an efficient bioadsorbent for the simultaneous removal of AO and ST.

For bicomponent adsorption of AO and ST on HDSB, the *R*^2^, *χ*^2^_red_, and *RSS* values (Table 4) for the AO isotherm suggested that the RAST equations (fed with the Sips and Langmuir fitting parameters) were more predictive, although the quality of the fit for the RAST-Sips model was slightly superior, as shown by higher *R*^2^ and lower *RSS* values. For bicomponent adsorption of ST and AO, all three models were predictive for the ST isotherm. These results were relevant for better understanding of the adsorption systems studied, since all the results discussed so far indicated that there was strong interaction between the two dye species in solution (Section 2.5) and, unlike the IAST model, this behavior is considered in the theoretical basis of the RAST model [42]. It could be seen from the plots of *q*_e,exp_ against *q*_e,est_ (Supplementary Figure S9a,b) that the experimental *q*_e_ values for AO adsorption were not well predicted by the IAST-Langmuir equation, while the RAST equations resulted in similar predictions for both dyes in the entire equilibrium concentration range investigated.

Such deviations from the “ideality” indicated by the high prediction capacity of the RAST model could be estimated from the plot of the experimental activity coefficient (*γ*) as a function of reduced spreading pressure (*ψ*), for simultaneous AO-ST adsorption on HDSB (Supplementary Figure S9c). All the *γ* values for both dyes were distinct and higher than unity (*γ*_AO_ ≠ *γ*_ST_ > 1), indicating a “non-ideal” adsorption behavior, where the experimental adsorption capacities tended to be lower than the corresponding values predicted for an “ideal” adsorption behavior [64,65].

The high *R*^2^ values (>0.9) for linear regression of the plot of *γ*_AO_ against *γ*_ST_ (*R*^2^ = 0.939) (Supplementary Figure S9d) supported application of the Wilson equation [66] to estimate fitting parameters related to the interaction effects between the two dye species (*Λ*_12_ and *Λ*_21_), completing the set of equations of the RAST model [64]. The much higher value of *Λ*_21_ than *Λ*_12_ (*Λ*_12_ = 7.871; *Λ*_21_ = 65.922) confirmed a large effect of ST adsorption on the adsorption of AO. The results further supported the existence of an antagonistic effect of ST on AO adsorption, reflecting the sharp decrease in the maximum adsorption capacity for AO in the presence of ST in the binary system, when compared to the monocomponent system.

Table 5 presents the *q*_e_ values for several (bio)materials used to remove AO and ST from aqueous solutions. Under similar conditions, the *q*_e_ values obtained in the present work were close to those reported by Fideles et al. [42] and lower than those found by Teodoro et al. [43]. This could be explained by the fact that pure cellulose was chemically functionalized to a greater extent than sugarcane bagasse, because access to the cellulose chains in the sugarcane bagasse was restricted by layers of lignin and hemicelluloses. Nevertheless, the use of sugarcane bagasse is a more attractive option, since this is an agricultural byproduct with low value and high availability. Furthermore, it presented higher *q*_e_ values than observed for several other biomasses (Table 5).

### 2.3. Adsorption Thermodymanics

**Figure 6:**
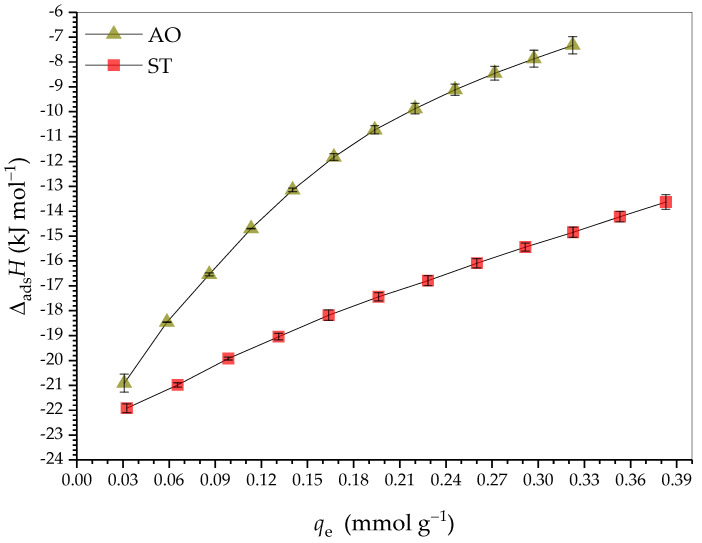
Curves of adsorption enthalpy change (Δ_ads_*H*) as a function of equilibrium adsorption capacity (*q*_e_) for monocomponent adsorption of AO and ST on HDSB at pH 7.0 and 25.0000 ± 0.0001 °C.

Figure 6 presents the curves of Δ_ads_*H* as a function of *q*_e_ for monocomponent adsorption of AO and ST on HDSB at pH 7.0 and 25.0 °C. Three independent subprocesses occurring during the adsorption could potentially contribute to Δ_ads_*H*, as shown in Equation (1):

(1) $${∆}_{ads}H/kJ\;{mol}^{-1}={∆}_{desol}H^{HDSB\;sites-dye}+{∆}_{int}H^{dye-dye}+{∆}_{int}H^{HDSB\;sites-dye}$$

where ${∆}_{desol}H^{HDSB\;sites-dye}$ (subprocess *i*) is the desolvation of the dye molecules and the adsorption sites on the surface of HDSB, ${∆}_{int}H^{dye-dye}$ (subprocess *ii*) is the formation of dye-dye interactions on the surface of HDSB and in the bulk solution, and ${∆}_{int}H^{HDSB\;sites-dye}$ (subprocess *iii*) is the formation of dye-HDSB binding site interactions. Subprocesses *i* and *ii* may be endothermic or exothermic, while subprocess *iii* is necessarily exothermic [67].

At the beginning of the curves of Δ_ads_*H* as a function of *q*_e_, where the dye concentration was very low, the contribution of the ${∆}_{int}H^{dye-dye}$ term could be disregarded. Since the ${∆}_{int}H^{HDSB\;sites-dye}$ term was always negative, for a negative value of Δ_ads_*H* to be obtained, the ${∆}_{desol}H^{HDSB\;sites-dye}$ term had to be positive (endothermic), but with lower magnitude than the ${∆}_{int}H^{HDSB\;sites-dye}$ term. As *q*_e_ increased, Δ_ads_*H* became less negative, because the dye concentration increased, so that subprocess *ii* (positive) became more significant. Whether occurring simultaneously or independently, subprocesses *i* and *iii* depended on the dye concentration and contributed to making the enthalpy value less negative, since the adsorption sites were energetically different and therefore led to different values for the ${∆}_{desol}H^{HDSB\;sites-dye}$ and ${∆}_{int}H^{HDSB\;sites-dye}$ terms, with higher energy binding sites probably being occupied first.

Since the adsorption of AO and ST on the HDSB binding sites was exothermic for the entire *q*_e_ range evaluated, increase in the surface coverage of HDSB resulted in the Δ_ads_*H* values becoming less negative, with Δ_ads_*H* values ranging from −21 to −7 kJ mol^−1^ for AO and from −22 to −14 kJ mol^−1^ for ST. Such increases of Δ_ads_*H* with increasing *q*_e_ were expected, due to the repulsive interactions between the positively charged dye molecules, making the ${∆}_{int}H^{dye-dye}$ term non-negligible and more positive as the bioadsorbent surface became more populated. Therefore, it could be inferred that the energy resulting from the three subprocesses *i*, *ii*, and *iii* depended on the HDSB surface coverage, becoming less exothermic as the adsorption occurred. In addition, this variation indicated that the HDSB binding sites were not uniform, in contrast to the Langmuir model assumption that all the binding sites are energetically equal. The results of the ITC experiments also suggested that the interaction of ST with HDSB involved higher energy binding sites, compared to AO. On the other hand, the Δ_ads_*H*/*dq*_e_ rate was higher for AO than ST.

Fideles et al. [42] observed that AO adsorption on sugarcane bagasse modified with trimellitic anhydride was more exothermic than ST adsorption, which was the opposite of the behavior observed in the present study. This difference suggested that the thermodynamics of adsorption of AO and ST on the surfaces of carboxylic acid-functionalized sugarcane bagasse depended on the type of carboxylic acid used (mono- or polycarboxylic, aliphatic or aromatic) and its physicochemical properties (including electronic effects, p*K*_a_ of the acid functions, and others).

The thermodynamic parameters for adsorption of AO and ST on HDSB were Δ_ads_*G*° = −23.1 ± 0.5 kJ mol^−1^ and −28.1 ± 0.8 kJ mol^−1^, Δ_ads_*H*° = −22.1 ± 0.3 kJ mol^−1^ and −23.44 ± 0.01 kJ mol^−1^, and *T*Δ_ads_*S*° = 0.9 ± 0.6 kJ mol^−1^ and 4.7 ± 0.8 kJ mol^−1^, respectively. The negative values for Δ_ads_*G*° and the magnitudes of the thermodynamic equilibrium constants (*K*_eq_ >> 1) (*K*_eq,AO_ = 10,944 ± 593 and *K*_eq,ST_ = 85,062 ± 9996) showed that under the standard conditions, the adsorption processes of AO and ST on HDSB were thermodynamically favorable. Hence, the dye molecules were preferentially adsorbed on the surface of HDSB, rather than remaining in their solvated forms in the bulk solution, and the equilibrium positions were closer to the products (HDSB-AO and HDSB-ST) than the reactants (AO or ST and HDSB). The magnitudes of the Δ_ads_*H*° and *T*Δ_ads_*S*° values showed that the adsorptions of AO and ST on HDSB were exothermic and enthalpically directed, under the standard conditions. Furthermore, although the Δ_ads_*H*° values indicated a predominance of physical mechanisms for adsorption of the dyes on HDSB (such as electrostatic, π-stacking, dipole–dipole, and van der Waals interactions), the experimental results suggested the involvement of both chemical and physical interactions, especially for ST. It is also necessary to consider that the Δ_ads_*H*° values included three contributions, with only one of them (${∆}_{int}H^{HDSB\;sites-dye}$) reflecting the interaction of the HDSB binding sites with the dye molecules.

In addition, the thermodynamic results showed that ST adsorption was more favorable than AO adsorption, probably involving different types and numbers of interactions (see Section 2.5), which could provide an explanation for the preferential adsorption of ST on HDSB in the bicomponent assays.

**Table 5:** Some adsorbent materials reported in the literature employed to remove AO and ST from water.

Adsorbent	Dye	*q*_e_ (mmol g^−1^)	pH	*T* (°C)	Particle Size (mm)	Dose (g L^−1^)	Reference
Cellulose modified with trimellitic anhydride	AO	2.63 ^1^	4.5	25	0.25	0.2	[43]
		5.18 ^1^	7.0				
Sugarcane bagasse modified with trimellitic anhydride	AO	1.01 ^1^	4.5	25	0.5	0.2	[42]
		1.73 ^1^	7.0				
Sugarcane bagasse ash	AO	0.12 ^2^	7.0	30	-	0.2	[68]
Activated carbon	AO	0.35 ^2^	7.0	25	-	0.04	[10]
Guava leaves	AO	0.03 ^2^	9.0	30	0.2	-	[69]
Sugarcane bagasse modified with phthalic and succinic anhydrides	AO	1.37 ^1^	7.0	25	0.5	0.2	This work
Sugarcane bagasse	AO	0.10 ^1^	7.0	25	0.5	0.2	This work
Nano Carbon/Polyurethane Tubes	ST	1.59 ^2^	7.0	30	(1.0–3.0) × 10^−5^	0.1	[70]
HDTMA-*algae*	ST	0.17 ^2^	4.0	25	9.3 × 10^−5^	5	[71]
Rice husks	ST	0.21 ^2^	6.5	40	0.15–0.3	2	[72]
Sugarcane bagasse modified with trimellitic anhydride	ST	0.64 ^1^	4.5	25	0.5	0.2	[42]
		1.23 ^1^	7.0	25			
Cellulose modified with trimellitic anhydride	ST	3.18 ^1^	4.5	25	0.25	0.2	[43]
		3.74 ^1^	7.0	25			
Sugarcane bagasse modified with phthalic and succinic anhydrides	ST	0.93 ^1^	7.0	25	0.5	0.2	This work
Sugarcane bagasse	ST	0.07 ^1^	7.0	25	0.5	0.2	This work

^1^ Results obtained from the equilibrium data. ^2^ Results obtained from the Langmuir model.

### 2.4. Evaluation of Reuse of the Hetero-Disubstituted Sugarcane Bagasse

For both dyes, higher desorption efficiencies (*E*_des,AO_ = ~43% and *E*_des,ST_ = ~54%; Table 6) were obtained using a time of 360 min. This was considered an appropriate desorption time, since no further satisfactory increase in *E*_des_ was obtained using longer times. Desorption was mainly attributed to an ion exchange mechanism, where the charged dye molecules were replaced by H^+^ ions (from H_3_O^+^) from the acid solution. Nevertheless, the incomplete desorption indicated that the acid solution was not able to disrupt all the HDSB-dye interactions, confirming the existence of other types of interactions, such as π interactions between the dye aromatic rings and phthalyl and lignin units in the HDSB structure. Similar results were reported by Teodoro et al. [43] and Fideles et al. [42].

Supplementary Table S2 presents the mass balances for the desorption and re-adsorption of AO and ST. Table 7 presents the re-adsorption capacities (*q*_e,re-adsorbed_) for AO and ST on HDSB after desorption. For ST, *q*_e,re-adsorbed_ was smaller than *q*_e_, while for AO, *q*_e_ was similar to *q*_e,re-adsorbed_. Since the *E*_des_ values were 31–56% (Table 6), while *E*_re-ad-AO_ = 99% and *E*_re-ad-ST_ = 77% (Table 7), this suggested that the non-desorbed dye molecules on the surface of HDSB could interact with other dye molecules in the bulk solution, increasing the total adsorption capacity of HDSB by acting as new adsorption sites. Although high values of *E*_re-ad_ were obtained (Table 7), the incomplete desorption indicated that strict control (monitoring) of the HDSB reuse operation would be required in wastewater treatment plants, because the values could vary after each new desorption/re-adsorption cycle.

### 2.5. Radial Distribution Functions

**Figure 7:**
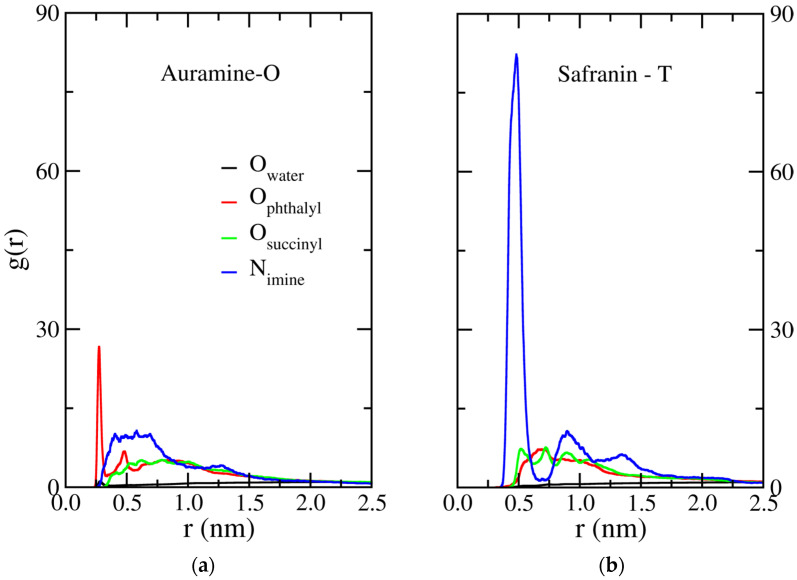
Pair radial distribution functions (RDFs) for (**a**) AO and (**b**) ST, considering the interactions of the *N*-imine of each dye with water oxygen (black lines), phthalyl carboxylate oxygen (red lines), succinyl carboxylate oxygen (green lines), and dye *N*-imine (blue lines).

As shown in Figure 7a, AO presented high structural correlations with other AO molecules and with the succinyl and phthalyl groups of the modified sugarcane bagasse cellulose. In the adsorption, the cationic imine groups of AO surrounded the phthalyl groups at regular distances, characterized by the peaks at 0.25 and 0.50 nm (red curves). In addition, low structural correlation between AO and water molecules indicated hydrophobic behavior. Figure 7b shows high structural correlations among the ST molecules and the modified sugarcane bagasse cellulose. This dye also showed low affinity with water molecules (higher hydrophobicity). An intense peak at 0.5 nm, together with peaks at 0.8 and 1.3 nm, indicated the formation of ST aggregates. In summary, the interactions profiles suggested that both dyes were preferentially adsorbed on the modified sugarcane bagasse cellulose, rather than interacting with water. The first peak of the AO radial distribution function (RDF) occurred near 0.3 nm, indicating short interaction distances (Figure 7).

## 3. Materials and Methods

### 3.1. Material

Phthalic anhydride (99.0%, MW = 148.12 g mol^−1^), succinic anhydride (98.0%, MW = 100.07 g mol^−1^), *N*,*N*-dimethylacetamide (chromatographic grade), auramine-O (99%, C.I. 41000, MW = 303.84 g mol^−1^), and safranin-T (99%, C.I. 50240, MW = 350.85 g mol^−1^) were purchased from Sigma-Aldrich (São Paulo, Brazil). Citric acid, monosodium citrate, disodium citrate, trisodium citrate, pyridine, acetone, ethanol (95% and 99.8%), hexane, anhydrous calcium chloride, hydrochloric acid (37 wt.% in water), nitric acid (65 wt.% in water), sodium nitrate, and sodium hydroxide were purchased from Synth (São Paulo, Brazil). Citrate buffer solutions (pH 2.13–7.14) were used to avoid pH variations during the adsorption experiments. All the aqueous solutions were prepared using ultrapure water (18.2 MΩ cm at 25 °C) obtained from a Milli-Q Simplicity^®^ system (Millipore, São Paulo, Brazil).

### 3.2. Preparation of Raw Sugarcane Bagasse and Hetero-Disubstituted Sugarcane Bagasse

Raw SB was kindly supplied by the Jatiboca plant (Urucânia, Minas Gerais, Brazil). The material was pretreated to remove water-soluble compounds, ground to reduce the particle size, and submitted to Soxhlet extraction with a mixture of hexane and ethanol to remove phenolic and other compounds, before chemical modification. These steps for preparation of the raw SB were performed as described by Ramos et al. [73].

Bi-functionalization to obtain HDSB employed a one-pot synthesis method. For this, 15.000 ± 0.001 g of SB, 51.371 ± 0.001 g of phthalic anhydride, 8.672 ± 0.001 g of succinic anhydride, and 225 mL of anhydrous pyridine (freshly distilled) were added (in this order) to a 500 mL round-bottomed flask. A reflux condenser coupled to a glass drying tube filled with powdered anhydrous calcium chloride was connected to the flask. The suspension was heated at 100 °C for 11 h in a canola oil bath, on a heating plate (PC-420D, Corning), under gentle agitation (300 rpm). After the heating period, the flask was removed from the canola oil bath and left to cool for 30 min. The mixture containing HDSB was poured into a glass Büchner funnel (500 mL, porosity 1), followed by rinsing under reduced pressure with 95% ethanol (500 mL), distilled water (750 mL), basic aqueous solution (0.01 mol L^−1^ NaOH, 750 mL), distilled water (750 mL), acid aqueous solution (0.01 mol L^−1^ HCl, 750 mL), distilled water (750 mL), 99.8% ethanol (500 mL), and acetone (500 mL). The HDSB was then dried to constant weight on a Petri dish in an oven (model 515 C, Fanem), at 85 °C, for 1.5 h. The weight gain (*wg*) was determined gravimetrically.

### 3.3. Characterization of Raw Sugarcane Bagasse and Hetero-Disubstituted Sugarcane Bagasse

Samples of SB and HDSB were characterized by point of zero charge (pH_PZC_) measurement, Fourier transform infrared spectroscopy (FT-IR), and ^13^C solid-state nuclear magnetic resonance (^13^C SS NMR). The specific surface areas and pore size distributions of SB and HDSB were also determined. Samples of HDSB loaded with AO or ST (HDSB-AO and HDSB-ST) were characterized by FT-IR and energy dispersive X-ray (EDX) spectroscopy. Before characterization, all the samples were dried in an oven at 85–90 °C for 1 h. Further details of the characterization procedures are provided in the Supplementary Material.

### 3.4. Batch Mono- and Bicomponent Adsorption Studies

The following standard experimental procedures were used in all the single and binary batch adsorption assays. All the adsorption assays were performed in triplicate. Buffered dye (AO and/or ST) solutions (100.00 mL) were added to 250 mL Erlenmeyer flasks and heated for 1 h at 25.0 ± 0.1 °C, in an orbital shaker-incubator (TE-424, Tecnal, Piracicaba, São Paulo, Brazil), under agitation at 130 rpm, followed by addition of the HDSB adsorbent. Further experimental conditions are shown in Table 8. The solid and liquid phases were separated by centrifugation for 10 min at 3600 rpm, using an Excelsa II 206 BL centrifuge (Fanem, Guarulhos, São Paulo, Brazil). The concentrations of AO (*λ*_max_ = 432 nm) and ST (*λ*_max_ = 530 nm) in the liquid phase were determined using a spectrophotometer (SP-220, Biospectro, Curitiba, Paraná, Brazil), with appropriate dilutions to obtain the analytical curves.

For bicomponent adsorption, the concentrations of AO and ST in the liquid phase were determined after appropriate dilution to obtain the analytical curves for AO and ST, measuring the absorbances *A*_432_ and *A*_530_ at wavelengths of 432 and 530 nm, corresponding to the maximum absorption bands of AO and ST, respectively. Analytical curves were constructed for pure AO and ST, with determination of the slopes (*k*) of the straight lines of the plots of the absorbances at 432 and 530 nm for AO and ST against the AO and ST concentrations (*k*_AO,432_ = 0.1138, *k*_ST,432_ = 0.0147, *k*_AO,530_ = 0.0000, and *k*_ST,530_ = 0.0942) [42,74]. The concentrations of AO and ST (*C*_y,dye_) in the liquid phases at the initial time (*y* = 0), at time *t* (*y* = *t*), and at equilibrium (*y* = *e*) were calculated using Equations (2) and (3):

(2) $$C_{y,AO}/mg\;L^{-1}=\left[\frac{\left(k_{ST,530}A_{432}\right)-\left(k_{ST,432}A_{530}\right)}{\left(k_{AO,432}k_{ST,530}\right)-\left(k_{AO,530}k_{ST,432}\right)}\right]df$$

(3) $$C_{y,ST}/mg\;L^{-1}=\left[\frac{\left(k_{AO,432}A_{530}\right)-\left(k_{AO,530}A_{432}\right)}{\left(k_{AO,432}k_{ST,530}\right)-\left(k_{AO,530}k_{ST,432}\right)}\right]df$$

where *df* is the dilution factor. Dye concentrations were converted to mmol L^−1^ based on the molecular weight of the cationic moiety of each dye molecule (MW_AO_^+^ = 268.34 g mol^−1^ and MW_ST_^+^ = 315.35 g mol^−1^).

The dye adsorption capacity (*q*_y,dye_) was calculated using Equation (4):

(4) $$q_{y,dye}/mmol\;g^{-1}=\frac{\left(C_{0,dye}-C_{y,dye}\right)V_{dye}}{w_{HDSB}}$$

where *V*_dye_ (L) is the volume of the dye (AO or ST) solution, *C*_0,dye_ (mmol L^−1^) is the dye (AO or ST) initial concentration, *C*_y,dye_ (mmol L^−1^) is the dye (AO or ST) concentration at time *t* or at equilibrium, and *w*_HDSB_ (g) is the weight of HDSB.

The dye removal (*R*_dye_) by HDSB was calculated using Equation (5) [44]:

(5) $$R_{dye}/\%=\left[\frac{\left(C_{0,dye}-C_{y,dye}\right)}{C_{0,dye}}\right]\times 100$$

### 3.5. Modeling of Single and Binary Batch Adsorption Data

The model equations used to fit the single and binary batch adsorption data are shown in Appendix A, Table A1, Table A2, Table A3 and Table A4, together with the error functions used to evaluate the quality of the model fits to the experimental data. Origin 2015 software (OriginLab Corp., Northampton, MA, USA) was used to estimate the model parameters for single adsorption, while Matlab 2010a (Mathworks Inc., Natick, MA, USA) was used to estimate the model parameters for binary adsorption. The computational procedures used to fit the binary adsorption data were those reported by Teodoro et al. [43] and Teodoro et al. [59].

### 3.6. Calculation of Adsorption Thermodynamic Parameters

The changes in the enthalpy of adsorption (∆_ads_*H*) as a function of HDSB surface coverage with AO or ST were obtained by isothermal titration calorimetry (ITC), using a nanocalorimeter (TAM III, TA Instruments, New Castle, DE, USA) equipped with two 4 mL stainless steel cells (reference and sample) and operated using TAM Assistant software. The ITC experiments were performed in duplicate, with the samples being degassed for 10 min before each experiment. Briefly, the buffered dye (AO or ST) solution (pH 7.0) was filled into a 500 µL Hamilton syringe controlled by a piston pump that added the dye solution stepwise into the calorimetric cell, which was previously filled with the buffer solution (pH 7.0). The sample cell contained buffer solution and HDSB, while the reference cell contained only buffer solution. A propeller stirrer was used to ensure homogeneity of the suspension in the sample cell. The ITC experimental conditions are provided in Table 9. The ∆_ads_*H* values were calculated using Equation (6):

(6) $${∆}_{ads}H/J\;{mol}^{-1}=\frac{\sum_{i=1}^{N}\left(q_{i,int}-q_{i,dil}\right)}{\sum_{i=1}^{N}n_{i}}$$

where *q_i_*_,int_ and *q_i_*_,dil_ are the energies in the form of heat absorbed or released after the *i*th injection of solute (AO or ST) solution into the cell with HDSB (sample cell) and the cell without HDSB (reference cell), respectively. The amount of solute adsorbed on HDSB after each injection, *n_i_* (mmol g^−1^), was estimated using the monocomponent adsorption isotherm for AO or ST on HDSB, as described by Pereira et al. [75].

The changes in the standard free energy of adsorption (*∆*_ads_*G*°) were calculated according to Equation (7):

(7) $${∆}_{ads}G{}^{\circ}/J\;{mol}^{-1}=-RTln\left[\frac{b}{\gamma_{e}}\left(1\;mol\;L^{-1}\right)\right]$$

where *R* is the ideal gas constant (8.314 J K^−1^ mol^−1^), *T* is the absolute temperature (K), *b* is the Langmuir constant (L mol^−1^), and *γ*_e_ is the activity coefficient at equilibrium at 25.0 °C [22].

According to Liu [76] and Ghosal and Gupta [77], the *γ*_e_ value can be calculated using the approximation proposed by Davies [78], according to Equation (8):

(8) $$\log\gamma_{e}=-0.5z^{2}\left(\frac{\sqrt{\mu }}{1+\sqrt{\mu }}-0.3\mu \right)$$

where *µ* (mol L^−1^) is the ionic strength of the solution at equilibrium and *z* is the ionic charge (+1 for AO and ST at pH 7.0) [42].

The changes in the standard entropy of adsorption (*∆*_ads_*S*°) were calculated using Equation (9), with the *∆*_ads_*H*° (J mol^−1^) value determined by ITC, as described by Pereira et al. [75].

(9) $${∆}_{ads}S/J\;K^{-1}{mol}^{-1}=\frac{{∆}_{ads}H{}^{\circ}-{∆}_{ads}G{}^{\circ}}{T}$$

### 3.7. Desorption and Re-Adsorption of the Hetero-Disubstituted Sugarcane Bagasse

HDSB was loaded with AO or ST, using the methodology described in Section 3.4 and the following experimental conditions: 0.0200 ± 0.0001 g of HDSB, 0.374 mmol L^−1^ aqueous AO or ST solution at pH 7.00, and agitation at 130 rpm and 25.0 ± 0.1 °C for 1800 min (AO) or 2460 min (ST).

After loading, HDSB-AO and HDSB-ST were recovered by single filtration, washed with an excess of ultrapure water, and dried in an oven at 80 °C for 4 h. Next, 100.0 mL volumes of 0.01 mol L^−1^ HCl were added to 250 mL Erlenmeyer flasks containing 0.0200 ± 0.0001 g of HDSB-AO or HDSB-ST, followed by stirring (130 rpm, 25.0 ± 0.1 °C) in an orbital shaker-incubator for different times (60, 180, and 360 min). The tests were performed in duplicate. The supernatants were collected, diluted, and the pH values were adjusted to the pH of the analytical curves for AO or ST (pH 7.0), using 0.01 mol L^−1^ NaOH solution. The desorption efficiency, *E*_des_ (%), was calculated as described by Teodoro et al. [43].

Evaluation was also made of the re-adsorption capacity of HDSB after one desorption test. The re-adsorption experimental conditions were as described in Section 3.4. For a single adsorption–desorption–re-adsorption cycle, the re-adsorption efficiency of HDSB, *E*_re-ad_ (%), was calculated as described by Fideles et al. [42] and Teodoro et al. [43].

### 3.8. Molecular Dynamics Simulations

**Figure 8:**
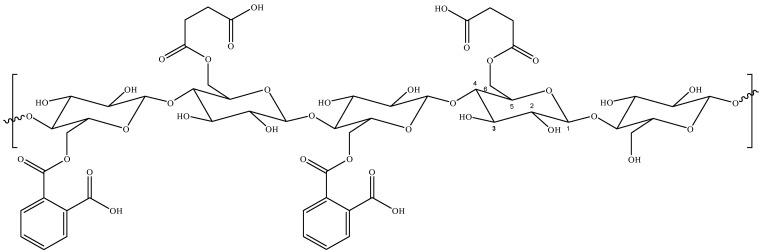
Proposed model structure for sugarcane bagasse cellulose modified with succinyl and phthalyl units (HDSB).

**Figure 9:**
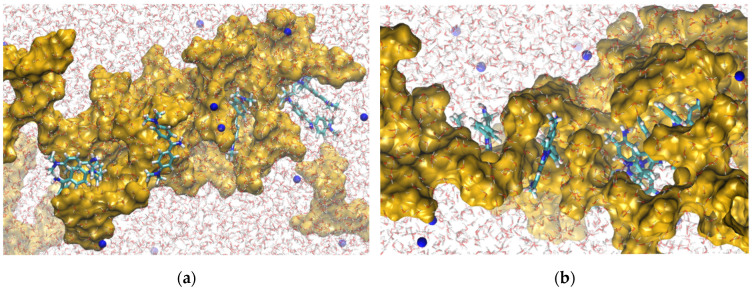
Snapshots of interactions between HDSB (yellow surface) and auramine-O (**a**), safranin-T (**b**), water (lines), and Na^+^ (blue spheres).

Molecular dynamics simulations with atomistic modeling were used to elucidate the interactions between AO and ST with HDSB (Figure 8). Firstly, the restrained electrostatic potential (RESP) charge parameters for all the components (the dyes and HDSB) were obtained from quantum mechanical calculations, employing density functional theory (DFT) at the PBE1PBE/6-31+G(d,p) level of theory. Implementation of the functional and basis set employed ORCA 4.2 software [79]. Subsequently, the data were inserted in the OPLS-AA force field [80] used in the simulations. Two boxes were constructed, with dimensions of 6.0 × 6.0 × 8.0 nm and periodic boundary conditions. Each box contained eight molecules of dye, twenty oligomers of modified sugarcane bagasse cellulose (HDSB), and water molecules to complete the volume. In addition, both systems were neutralized by adding sodium atoms (Figure 9).

The simulations were performed maintaining constant temperature (300 K) and pressure (1.0 bar). The gradient descent method was used in the minimization stages, to avoid strong repulsive contacts between the atoms of neighboring molecules. In the equilibration stages, the NVT and NPT ensembles were employed during 10.0 ns, with a time step of 2 fs, to adjust the configurational arrangements in the box. The production stages were performed in 100 ns. The neighboring list for the calculation of nonbonded interactions was updated every 10 time steps, with a cut-off of 1.2 nm. The same cut-off was used for the Lennard–Jones potential. The electrostatic calculations employed the Particle-Mesh Ewald (PME) method [81,82]. These simulation protocols and the parameters were based on procedures described in the literature [83,84]. All the minimization steps and the molecular dynamics simulations employed GROMACS 2019 software [85,86].

## 4. Conclusions

The HDSB bioadsorbent produced from sugarcane bagasse (SB) by simultaneous chemical modification with phthalic and succinic anhydrides provided the efficient removal of auramine-O (AO) and safranin-T (ST) from spiked aqueous solutions, exhibiting a maximum adsorption capacity (*Q*_max_) about 10-fold higher than obtained for SB. The chemical modifications made HDSB more selective, which resulted in longer equilibrium times for the adsorption of AO and ST on HDSB than on SB. For both dyes, *Q*_max_ occurred at pH 7.0, with the HDSB binding sites exhibiting higher affinity for ST than AO in both mono- and bicomponent systems. The adsorption of the dyes in the bicomponent system showed antagonistic behavior, with the presence of ST suppressing AO adsorption. Calorimetric measurements confirmed the heterogeneity of the binding sites on the surface of HDSB and the existence of different interactions between the HDSB binding sites and the dyes. The results for both ST and AO were suggestive of preferential adsorption at specific binding sites (phthalyl units), while ST also showed high capacity to become structured at short distances around succinyl units. Under standard conditions, the adsorption of AO and ST was exergonic and exothermic (enthalpically driven). The acid desorption solution was not able to remove all the AO and ST molecules adsorbed on HDSB, but it was possible to use HDSB in a new adsorption cycle, without a great loss of *Q*_max_, especially in the case of AO.

## 5. Patents

There is a patent deposited resulting from the work reported in this manuscript assigned as BR 102017013780-5 A2.

## Figures and Tables

**Table 1:** Results of nonlinear regression analysis of the monocomponent experimental kinetic data for dyes adsorption on HDSB and SB (*C*_0_ = 0.374 mmol L^−1^ AO or ST, 0.2 g L^−1^ adsorbent, 130 rpm, 25 °C, and pH 7.0).

Parameter	HDSB		SB	
	AO	ST	AO	ST
*q*_e_,_exp_ (mmol g^−1^)	1.008 ± 0.006	0.862 ± 0.001	0.140 ± 0.000	0.061 ± 0.001
*t*_e_ (min)	1800	2460	360	900
Pseudo-first order (PFO)				
*q*_e,est_ (mmol g^−1^)	0.82 ± 0.05	0.74 ± 0.03	0.135 ± 0.003	0.062 ± 0.004
*k*_1_ (min^−1^)	(9 ± 2) × 10^−3^	(1.1 ± 0.2) × 10^−2^	(2.15 ± 0.04) × 10^−1^	(8 ± 1) × 10^−3^
*R* ^2^	0.812	0.748	0.558	0.961
*R* ^2^_adj_	0.799	0.736	0.469	0.954
χ^2^_red_	2.4 × 10^−2^	2.1 × 10^−2^	3.0 × 10^−4^	9.0 × 10^−4^
Pseudo-second order (PSO)				
*q*_e,est_ (mmol g^−1^)	0.93 ± 0.05	0.79 ± 0.03	0.138 ± 0.002	0.069 ± 0.007
*k*_2_ (g mmol^−1^min^−1^)	(1.3 ± 0.3) × 10^−2^	(2.4 ± 0.5) × 10^−2^	4.1 ± 0.9	0.11 ± 0.04
*R* ^2^	0.911	0.881	0.850	0.931
*R* ^2^_adj_	0.905	0.875	0.820	0.918
*χ* ^2^_red_	1.1 × 10^−2^	1.0 × 10^−2^	9.0 × 10^−5^	1.6 × 10^−3^
Elovich				
*t*_0_ (min)	21 ± 5	13 ± 5	(2.78 ± 0.00) ×10^−14^	(8.64 ± 0.00) × 10^−13^
*α* (mmol g^−1^min^−1^)	(1.8 ± 0.3) × 10^−2^	(3.78 ± 0.73) × 10^−2^	(8.3 ± 0.3) ×10^−8^	(1.1 ± 0.3) × 10^−3^
*β* (g mmol^−1^)	5.2 ± 0.2	7.6 ± 0.3	(23 ± 3) × 10^1^	(7 ± 1) × 10^1^
*R* ^2^	0.994	0.992	0.933	0.913
*R* ^2^_adj_	0.993	0.991	0.899	0.869
*χ* ^2^_red_	8.0 × 10^−4^	7.0 × 10^−4^	5.0 × 10^−5^	2.5 × 10^−3^
Intraparticle diffusion (IPD)				
*Step 1*				
*k*_d,1_ (mmol g^−1^ min^−1/2^)	(2.9 ± 0.2) × 10^−2^	(2.7 ± 0.1) × 10^−2^	(1.6 ± 0.3) × 10^−3^	(5.5 ± 0.8) × 10^−3^
*C* (mmol g^−1^)	(1.6 ± 0.3) × 10^−1^	(1.7 ± 0.1) × 10^−1^	(1.2 ± 0.0) × 10^−1^	−(2.0 ± 0.8) × 10^−2^
*R* ^2^	0.975	0.991	0.944	0.960
*R* ^2^_adj_	0.969	0.989	0.916	0.939
*Step 2*				
*k*_d,2_ (mmol g^−1^ min^−1/2^)	(1.3 ± 0.0) × 10^−2^	(6.8 ± 0.3) × 10^−3^	-	-
*C*	(4.6 ± 0.1) × 10^−1^	(5.2 ± 0.1) × 10^−1^	-	-
*R* ^2^	0.994	0.979		
*R* ^2^_adj_	0.992	0.977	-	-
Boyd plot				
*B*	(1.9 ± 0.1) × 10^−3^	(2.4 ± 0.1) × 10^−3^	(9 ± 1) × 10^−3^	(9.2 ± 0.7) × 10^−4^
*D_i_* (m^2^ min^−1^)	1.17 × 10^−11^	1.50 × 10^−11^	6.00 × 10^−11^	5.84 × 10^−11^

**Table 2:** Results of nonlinear regression analysis of the bicomponent experimental kinetic data for dyes adsorption on HDSB (C_0_ = 0.374 mmol L^−1^ AO or ST, 0.2 g L^−1^ adsorbent, 130 rpm, 25 °C, and pH 7.0).

Parameter	AO	ST
*q*_e_,_exp_ (mmol g^−1^)	0.672 ± 0.006	1.242 ± 0.004
*t*_e_ (min)	1980	2160
Pseudo-first order (PFO)		
*q*_e,est_ (mmol g^−1^)	0.55 ± 0.03	1.05 ± 0.03
*k*_1_ (min^−1^)	0.013 ± 0.003	0.07 ± 0.01
*R* ^2^	0.760	0.567
*R* ^2^_adj_	0.748	0.545
χ^2^_red_	0.019	0.014
Pseudo-second order (PSO)		
*q*_e,est_ (mmol g^−1^)	0.59 ± 0.02	1.09 ± 0.02
*k*_2_ (g mmol^−1^ min^−1^)	0.028 ± 0.006	0.09 ± 0.02
*R* ^2^	0.873	0.752
*R* ^2^_adj_	0.866	0.739
*χ* ^2^_red_	0.010	0.008
Corsel		
*α*	0.170	6.668
*β*	0.529	1.954
*γ* (*α + β*)	0.700	8.622
*γ_i_* − *γ_j_*	−7.921	
*Γ*_max_ (mmol m^−2^)	0.125	0.195
*k_+_*_1_ (L mmol^−1^ min^−1^)	74.3	710
*k_−_*_1_ (min^−1^)	8.938	8.209
*K = k_+_*_1_*/k*_−1_ (L mmol^−1^)	8.314	86.5
*D_s_* (m^2^ min^−1^)	1.17 × 10^−11^	1.50 × 10^−11^
*R* ^2^	0.300	0.315
*χ* ^2^_red_	0.010	0.003

**Table 3:** Results of nonlinear regression analysis for modeling the equilibrium adsorption data with monocomponent isotherm models (25 °C, 130 rpm, pH 7.0, and 0.2 g L^−1^ adsorbent).

Parameter	HDSB		SB	
	AO	ST	AO	ST
*Q*_max,exp_ (mmol g^−1^)	1.362 ± 0.009	0.929 ± 0.003	0.1042 ± 0.0009	0.074 ± 0.002
*I*_e_ (mol L^−1^)	0.1507	0.1507	-	-
*γ*_e_	0.7593	0.7593	-	-
Langmuir				
*Q*_max,est_ (mmol g^−1^)	1.72 ± 0.03	0.93 ± 0.03	0.21 ± 0.03	0.085 ± 0.004
*b* (L mmol^−1^)	8.3 ± 0.4	65 ± 8	2.3 ± 0.5	20 ± 2
*K*_eq_	10,944 ± 593	85,062 ± 9996	-	-
*R* ^2^	0.9950	0.9950	0.9752	0.9857
*R* ^2^_adj_	0.9947	0.9708	0.9734	0.9845
*χ* ^2^_red_	0.0017	0.0048	0.0007	0.0003
*RSS*	0.0273	0.0533	0.0100	0.0043
Sips				
*Q*_max,est_ (mmol g^−1^)	1.64 ± 0.06	0.88 ± 0.03	0.15 ± 0.08	0.096 ± 0.009
*b* (L mmol^−1^)	9.4 ± 0.9	76 ± 8	3 ± 3	14 ± 4
*n*	0.93 ± 0.05	0.8 ± 0.1	1.0 ± 0.2	1.2 ± 0.1
*R* ^2^	0.9959	0.9785	0.9411	0.9893
*R* ^2^_adj_	0.9953	0.9741	0.9321	0.9874
*χ* ^2^_red_	0.0016	0.0043	0.0018	0.0003
*RSS*	0.0243	0.0779	0.0040	0.0032
Redlich–Peterson (R-P)				
*K*_R_ (L g^−1^)	14 ± 1	60 ± 9	0.4 ± 0.1	1.9 ± 0.3
*a*_R_ (L mmol^−1^)	8.3 ± 0.6	65 ± 9	1.4 ± 0.6	21 ± 3
*Q*_max,est_ (mmol g^−1^)	1.7 ± 0.2	0.9 ± 0.2	0.3 ± 0.1	0.09 ± 0.02
*β*	1.00 ± 0.06	1.00 ± 0.04	1.0 ± 0.9	0.92 ± 0.07
*R* ^2^	0.9953	0.9732	0.9692	0.9871
*R* ^2^_adj_	0.9946	0.9679	0.964	0.9843
*χ* ^2^_red_	0.0019	0.0018	0.0095	0.0004
*RSS*	0.0279	0.0533	0.0024	0.0018
Dubinin–Radushkevich (D-R)				
*Q*_max,est_ (mmol g^−1^)	1.61 ± 0.02	1.01 ± 0.05	0.12 ± 0.01	0.088 ± 0.003
*k* (mmol^2^ kJ^−2^)	0.0202 ± 0.0005	0.0079 ± 0.0008	0.0284 ± 0.003	0.0133 ± 0.0006
*R* ^2^	0.9949	0.9383	0.9328	0.9867
*R* ^2^_adj_	0.9945	0.9326	0.9280	0.9870
*χ* ^2^_red_	0.0019	0.0112	0.0019	0.0003
*RSS*	0.0304	0.1231	0.0273	0.0040

**Table 4:** Results of adsorption equilibrium data modeling with IAST and RAST models for bicomponent AO-ST adsorption on HDSB (25 °C, 130 rpm, pH 7.0, and 0.2 g L^−1^ adsorbent).

Type of Dye	Model-Isotherm	*RSS*	*χ* ^2^_red_	*R* ^2^
AO	IAST-Langmuir	0.122	0.013	0.777
	RAST-Sips	0.012	0.003	0.964
	RAST-Langmuir	0.022	0.004	0.959
ST	IAST-Langmuir	0.019	0.003	0.910
	RAST-Sips	0.017	0.002	0.920
	RAST-Langmuir	0.018	0.002	0.913

**Table 6:** Desorption efficiency (***E*_des_**/%) of AO and ST dyes from HDSB at different contact times (0.01 mol L^−1^ HCl, 130 rpm, and 25 °C).

Dye	Desorption Time (min) ^1^		
	60	180	360
AO	32.3 ± 0.9	38.6 ± 0.2	42.8 ± 0.2
ST	40 ± 1	51 ± 1	54 ± 2

^1^ Desorption times greater than 360 min were investigated but there was no increase in *E*_des_.

**Table 7:** Evaluation of re-adsorption capacities of AO and ST on HDSB after the desorption step.

Dye	*q*_e,initial_ (mmol g^−1^)	*q*_e,re-adsorbed_ (mmol g^−1^)	*E*_re-ad_ (%)
AO	0.97 ± 0.01	0.96 ± 0.08	99
ST	0.71 ± 0.02	0.55 ± 0.07	77

**Table 8:** Summary of the experimental conditions used in the single and binary batch adsorption experiments of auramine-O (AO) and safranin-T (ST) on hetero-disubstituted sugarcane bagasse (HDSB).

Parameter	Type of Single Adsorption Experiment						Type of Binary Adsorption Experiment		
	Adsorbent Dosage	Solution pH	Contact Time		Initial Dye Concentration		Solution pH	Contact Time	Initial Dye Concentration
	AO or ST	AO or ST	AO	ST	AO	ST	AO-ST		
Initial pH	7.00	3.13–7.14 ^2^	7.00	7.00	7.00	7.00	3.13–7.14 ^2^	7.00	7.00
Weight of HDSB (g)	0.0100–0.0800 (±0.0001)	0.0200 (±0.0001)					0.0200 (±0.0001)		
Dye concentration (mmol L^−1^)	0.374				0.037–0.845	0.033–0.834	0.374 ^1^		0.037–0.894 (AO)/0.045–1.085 (ST) ^1^
Agitation time (min)	1440	1440	10–2160	10–2760	1800	2460	1440	10–2700	2280

^1^ Experiments were carried out using equimolar concentrations of AO and ST. ^2^ Buffered solutions used were 0.05 mol L^−1^ citric acid/monosodium citrate (pH 3.13), 0.05 mol L^−1^ monosodium citrate/disodium citrate (pH 4.13–5.13), and 0.05 mol L^−1^ disodium citrate/trisodium citrate (pH 6.13–7.14).

**Table 9:** Experimental conditions used in the isothermal titration calorimetry.

Parameter	Dye	
	AO	ST
Initial dye concentration in the syringe (mg L^−1^)	282.2	336.9
Solution pH ^1^	7.00	
Temperature (°C)	25.0000 ± 0.0001	
Agitation speed (rpm)	180	
Initial volume of buffer in the calorimetric cells (mL)	2.70 ± 0.01	
Weight of HDSB in the sample calorimetric cell (g)	0.00800 ± 0.00001	
Injection volume (µL)	40	
Interval of time between two consecutive injections (min)	35	

^1^ Buffer solution (citric acid/sodium citrate buffer).

## Data Availability

The raw data supporting the conclusions of this article will be made available by the authors on request.

## Supplementary Materials

The following supporting information can be downloaded at: https://www.mdpi.com/article/10.3390/molecules30153163/s1, Figure S1: Micrographs of surface mapping of nitrogen of HDSB loaded with (a) AO, (b) ST, and (c) AO and ST, at 100× magnification obtained by MEV-EDX; Figure S2: Effect of HDSB dosage on (a) AO and (b) ST adsorption (0.374 mmol L^−1^ AO or ST, 130 rpm, 25.0 ± 0.1 °C, 24 h, pH 7.0); Figure S3: Curves of equilibrium pH (pH_e_) versus HDSB weight percentage; Figure S4: Distribution curves of species of (a) ST and (b) AO, as a function of pH (curves were built using *pK*_a_ values of 9.8 and 6.4 for AO and ST, respectively); Figure S5: IPD plots for adsorption of (a) AO and (b) ST on HDSB (0.374 mmol L^−1^ AO or ST, 0.2 g L^−1^ HDSB, 130 rpm, 25.0 ± 0.1 °C, pH 7.0); Figure S6: IPD plots for adsorption of (a) AO and (b) ST on SB (0.374 mmol L^−1^ AO and ST, 0.2 g L^−1^ SB, 130 rpm, 25.0 ± 0.1 °C, pH 7.0); Figure S7: Boyd plot for adsorption of (a) AO and (b) ST on HDSB (0.374 mmol L^−1^ AO or ST, 0.2 g L^−1^ HDSB, 130 rpm, 25.0 ± 0.1 °C, pH 7.0); Figure S8: Boyd plot for adsorption of (a) AO and (b) ST on SB (0.374 mmol L^−1^ AO or ST, 0.2 g L^−1^ SB, 130 rpm, 25.0 ± 0.1 °C, pH 7.0); Figure S9: Experimental (*q*_e,exp_) and estimated (*q*_e,est_) equilibrium adsorption capacities for (a) AO and (b) ST on HDSB for IAST and RAST models; (c) experimental activity coefficients (*γ*) for AO and ST, as function of reduced spreading pressure (*ψ*); and (d) experimental *γ*_ST_ against experimental *γ*_AO_ (0.2 g L^−1^ HDSB, pH 7.0, 25.0 ± 0.1 °C, 130 rpm); Table S1: Mass balance for the desorption and re-adsorption experiments. [87,88,89,90,91,92,93].

## Abbreviations

The following abbreviations are used in this manuscript:

AO	Auramine-O
*b*	Langmuir constant (L mol^−1^)
*B*	Boyd plot slope
*C_i_*_,AO_	Auramine-O solution concentration at time *t* (*x* = *t*) or equilibrium (*x* = *e*) (mmol L^−1^)
*C_i_*_,ST_	Safranin-T solution concentration at time *t* (*x* = *t*) or equilibrium (*x* = *e*) (mmol L^−1^)
∆_ads_*G*°	Change in standard free energy of adsorption (kJ mol^−1^ or J mol^−1^)
∆_ads_*H*	Change in enthalpy of adsorption (kJ mol^−1^ or J mol^−1^)
∆_ads_*H*°	Change in standard enthalpy of adsorption (kJ mol^−1^ or J mol^−1^)
Δ_ads_*S*°	Change in standard entropy of adsorption (J K^−1^ mol^−1^)
*D*_s_	Surface diffusion coefficient (m^2^ min^−1^) of the Corsel model
*D*_i_	Effective diffusion coefficient of species *i* (m^2^ min^−1^) of the Boyd model
*E*_des_	Desorption efficiency (%)
*E*_re-ads_	Re-adsorption efficiency (%)
HDSB	Hetero-disubstituted sugarcane bagasse
HDSB-AO	Hetero-disubstituted sugarcane bagasse loaded with AO
HDSB-ST	Hetero-disubstituted sugarcane bagasse loaded with ST
IAST	Ideal Adsorbed Solution Theory
IPD	Intraparticle diffusion model
*k*_,di_	Intraparticle diffusion rate constant (mmol g^−1^ min^−1/2^) of step *i*
*µ*	Ionic strength (mol L^−1^)
*n*	Sips parameter (heterogeneity of the adsorption system)
pH_PZC_	Point of zero charge
*q*_e_	Equilibrium adsorption capacity (mmol g^−1^)
*Q*_max_	Maximum adsorption capacity (mmol g^−1^)
*q*_x,AO_	Adsorption capacity at time *t* (x = *t*) or at equilibrium (x = *e*) of AO on HDSB (mmol g^−1^)
*q*_x,ST_	Adsorption capacity at time *t* (x = *t*) or at equilibrium (x = *e*) of ST on HDSB (mmol g^−1^)
*R*	Gas constant (J K^−1^ mol^−1^)
*R*^2^	Coefficient of determination
*R*^2^_adj_	Adjusted coefficient of determination
RAST	Real Adsorbed Solution Theory
RDF	Radial Distribution Function
*RSS*	Residual sum of squares
ST	Safranin-T
SB	Sugarcane bagasse
*T*	Temperature (K or °C)
*t*	Time
*T*Δ_ads_*S*°	Entropic contribution (kJ mol^−1^)
*V*_dye_	Volume (L) of the dye (AO or ST)
*wg*	Weight gain (%)
*w*_HDSB_	Weight of HDSB (g)
*γ*_e_	Activity coefficient at equilibrium
*χ*^2^_red_	Reduced chi-square

## Appendix A

**Table A1:** Kinetics model equations used to fit single batch adsorption data.

Kinetic Model	Model Equation	Parameter	Reference
Pseudo-first order (PFO)	$q_{t}=q_{e}\left[1-\exp\left(-k_{1}t\right)\right]$	*k*_1_ (min^−1^): pseudo-first order rate constant	[50]
	$h=k_{1}q_{e}$	*h* (mmol g^−1^min^−1^): initial adsorption rate	
Pseudo-second order (PSO)	$q_{t}=\frac{k_{2}{q_{e}}^{2}t}{1+k_{2}q_{e}t}$	*k*_2_ (g mmol^−1^ min^−1^): pseudo-second order rate constant	[51]
	$h=k_{2}q_{e}^{2}$	*h* (mmol g^−1^min^−1^): initial adsorption rate	
Elovich	$q_{t}=\frac{1}{\beta }\ln\left(1+\alpha \beta \right)+\frac{1}{\beta }\ln\left(t+t_{0}\right)$	*α* (mmol g^−1^ min^−1^): initial adsorption rate *β* (g mmol^−1^): desorption rate constant.	[52]
Intraparticle diffusion (IPD)	$q_{t}=k_{di}t^{\frac{1}{2}}+C$	*k*_i_ (mmol g^−1^min^−1/2^): intraparticle diffusion rate constant *C* (mmol g^−1^): model constant	[53]
Boyd	$B_{t}={\left(\sqrt{\pi }-\sqrt{\pi -\frac{\pi^{2}f}{3}}\right)}^{2}for\;f<0.85$	*f* (*f = q*_t_ *q*_e_^−1^): fractional surface coverage	[54]
	$B=\frac{\pi^{2}D_{i}}{r^{2}}$	*D_i_* (m^2^ min^−1^): effective diffusion coefficient	

**Table A2:** Isotherm model equations and error function used to fit single batch adsorption data.

Isotherm Model	Model Equation	Parameter	Reference
Langmuir	$q_{e}=\frac{Q_{max}{bC}_{e}}{1+bC_{e}}$	*Q*_max_ (mmol g^−1^): maximum adsorption capacity *b* (L mmol^−1^): Langmuir binding constant	[60]
Sips	$q_{e}=Q_{max}\frac{{\left(bC_{e}\right)}^{\frac{1}{n}}}{1+{\left(bC_{e}\right)}^{\frac{1}{n}}}$	*n* (dimensionless): heterogeneity of the adsorption system	[61]
Redlich–Peterson (R-P)	$q_{e}=\left(\frac{K_{R}C_{e}}{1+a_{R}C_{e}^{\beta }}\right)$	*K*_R_ (L g^−1^): model constant *a*_R_ (L mmol^−1^): model constant *β* (dimensionless): model exponent	[62]
Dubinin–Radushkevich (D-R)	$q_{e}=q_{s}\exp\left(-B\varepsilon^{2}\right)$ with $\varepsilon =RT\ln\left(1+\frac{1}{C_{e}}\right)$ $E=\frac{1}{\sqrt{2B}}$	*q*_s_ (mmol g^−1^): maximum adsorption capacity *B* (mol^2^ kJ^−2^): constant related to the adsorption energy *ɛ* (kJ mol^−1^): Polanyi potential *R*: gas constant (8.314 J K^−1^ mol^−1^) *T* (K): temperature *E* (kJ mol^−1^): adsorption energy	[63]
Error function			
Reduce Chi-square (*χ*^2^_red_)	$\chi_{\mathrm{red}}^{2}=\frac{\sum_{i=1}^{N}w_{i}{\left(y_{i}-{\hat{y}}_{i}\right)}^{2}}{\upsilon }$	*w*_i_: weighting coefficient υ (υ = *N* − *P*): number of degrees of freedom *N*: number of experimental data points *P*: number of variables of the model	[42]

**Table A3:** Kinetics model equations and error function used to fit the binary batch adsorption data.

Kinetic Model	Model Equation	Parameter	Reference
Corsel	$\frac{d\Gamma (t)}{dt}=k(\Gamma {)}_{\mathrm{on}}^{\mathrm{app}}\left(\Gamma_{\max}-\Gamma \left(t\right)\right)C_{b}-k(\Gamma {)}_{\mathrm{off}}^{\mathrm{app}}\Gamma (t)$ with $k(\Gamma {)}_{\mathrm{on}}^{\mathrm{app}}=\frac{k(\Gamma {)}_{\mathrm{on}}^{\mathrm{int}}D_{s}}{\left[D_{s}+\delta k(\Gamma {)}_{\mathrm{on}}^{\mathrm{int}}(\Gamma_{\max}-\Gamma )\right]}$ $k(\Gamma {)}_{\mathrm{off}}^{\mathrm{app}}=\frac{k(\Gamma {)}_{\mathrm{off}}^{\mathrm{int}}D_{s}}{\left[D_{s}+\delta k(\Gamma {)}_{\mathrm{off}}^{\mathrm{int}}(\Gamma_{max}-\Gamma )\right]}$ and $k(\Gamma {)}_{\mathrm{on}}^{\mathrm{app}}=k_{+1}\exp(-\alpha \Gamma )$ $k(\Gamma {)}_{\mathrm{off}}^{\mathrm{app}}=k_{-1}\exp(+\beta \Gamma )$ For a binary system: $\;\Gamma =\Gamma_{i}+\Gamma_{j}$ $K_{a}=\frac{k_{\mathrm{on}}^{\mathrm{int}}}{k_{\mathrm{off}}^{\mathrm{int}}}$ $K_{a}=\frac{k_{\mathrm{on}}^{\mathrm{int}}}{k_{\mathrm{off}}^{\mathrm{int}}}=K\exp(-\gamma \Gamma )$ with γ=α+β and $K=\frac{k_{+1}}{k_{-1}}$	*Γ*(t) (mmol m^−2^): concentration of dye on the adsorbent surface *Γ*_max_ (mmol m^−2^): maximum surface coverage *k*(*Γ*)_on_ (L mmol^−1^ min^−1^): adsorption rate function *k*(*Γ*)_off_ (min^−1^): desorption rate function *C*_b_ (mmol L^−1^): dye concentration in the bulk *D*_s_ (m^2^ min^−1^): Surface diffusion coefficient *δ* (m): unstirred layer thickness *k*_+1_ (L mmol^−1^min^−1^): initial intrinsic rate constant *k*_−1_ (min^−1^): initial intrinsic desorption rate constants *α*: adsorption interaction constant *β*: desorption interaction constant *K*: association constant	[55]

**Table A4:** Isotherm model equations and error function used to fit the binary batch adsorption data.

Isotherm Model	Model Equation	Parameter	Reference
Real Adsorbed Solution Theory (RAST)	$C_{e}=C_{e}^{0}(T,P,\Psi )x_{i}\gamma_{i},\;i=1,2,\ldots ,\;N$ $\frac{1}{q_{T}}=\frac{x_{i}}{q_{e,i}^{0}}+\frac{x_{j}}{q_{e,j}^{0}}\;with\;x_{T}=x_{i}+x_{j}=1$ $\begin{array}{cc} \ln\gamma_{i} & =\left[1-\exp(-c\Psi )\right]\\ & \times \left[1-\ln\left(x_{i}+x_{j}\Lambda_{ij}\right)-\left(\frac{x_{i}}{x_{i}+x_{j}\Lambda_{ij}}\right)-\left(\frac{x_{j}\Lambda_{ji}}{x_{i}\Lambda_{ji}+x_{j}}\right)\right] \end{array}$ $C_{0,i}=[C_{e,i}^{0}+\frac{w}{V}(\sum_{j=1}^{N}\frac{x_{j}}{q_{e,i}^{0}})]x_{i}\;with\;C_{e,i}=C_{e,i}^{0}x_{i}$ $q_{e,i}=x_{i}q_{T}={\left(\sum_{j=1}^{N}\frac{x_{j}}{q_{e,i}^{0}}\right)}^{-1}$ For RAST model fed with the Langmuir isotherm: $\psi_{i}=\frac{\Pi_{i}A}{RT}=\int_{0}^{C_{e,i}^{0}}\frac{q_{e,i}^{0}\left(C_{e,i}^{0}\right)}{C_{e,i}^{0}}dC_{e,i}^{0}=Q_{\max,i}\ln\left(1+b_{i}C_{e,i}^{0}\right)$ For RAST model fed with the Sips isotherm: $\psi_{i}=\frac{\Pi_{i}A}{RT}=\int_{0}^{C_{e,i}^{0}}\frac{q_{e,i}^{0}\left(C_{e,i}^{0}\right)}{C_{e,i}^{0}}dC_{e,i}^{0}=\left(\frac{Q_{\max,i}}{n_{i}}\right)\ln\left[1+{\left(b_{i}C_{e,i}^{0}\right)}^{n_{i}}\right]$ Approximation proposed by Erto et al. [64]: $\gamma_{j}=\frac{a}{x_{i}}-\frac{bx_{j}}{x_{i}}\gamma_{i}\;with\;a=\frac{C_{T}}{C_{e,i}^{0}}\;and\;b=\frac{C_{e,j}^{0}}{C_{e,i}^{0}}$ $\frac{C_{e,j}^{0}}{C_{e,i}^{0}};\frac{q_{e,j}^{0}}{q_{e,i}^{0}}=\mathrm{constant}\;and\;\frac{q_{e,j}^{0}}{q_{e,j}^{0}+q_{e,i}^{0}}=x_{j}=\mathrm{constant}$	*N*: number of dyes in the mixture *C*_e_ (mmol L^−1^): dye equilibrium concentration in the liquid phase *C*^0^_e,*i*_ (mmol L^−1^): dye concentration in the liquid phase in equilibrium with dye concentration adsorbed on the solid phase [*q*^0^_e,*i*_ (mmol g^−1^] *T*: temperature *P*: pressure *Ψ*: reduced spreading pressure *γ_i_*: adsorbed dye activity coefficient *x_i_*: adsorbed dye mole fraction *q*_T_ (mmol g^−1^): total dye solid phase concentration *V* (L): volume *w* (g): adsorbent weight *c*: adjustable model parameter *Λ_ij_*: interaction effect parameter *C*_T_ (mmol L^−1^): total solute concentration *x_i_* = mole fraction of the species *i* *a* and *b*: adjustment constants of the approximation proposed by Erto et al. [64]	[64]
Error function			
Residual Sum of Squares (*RSS*)	$RSS=\sum_{i=1}^{N}{\left(y_{i}-{\hat{y}}_{i}\right)}^{2}$	*y*_i_: experimental data point *ŷ*_i_: estimated data point	[42]
