# Supplementary material for: Hetero-Disubstituted Sugarcane Bagasse as an Efficient Bioadsorbent for Cationic Dyes
Source: Molecules. 2025 Jul 29;30(15):3163. doi: 10.3390/molecules30153163 (PMC12348627; doi:10.3390/molecules30153163)
Supplement: Supplementary file 1 [file molecules-30-03163-s001.zip › molecules-3709806-supplementary.pdf]

Hetero-disubstituted sugarcane bagasse as an efficient bioadsorbent for cationic dyes

Megg Madonyk Cota Elias [1], Liliane Catone Soares [1], Oscar Fernando Herrera Adarme [2], Gabriel Max Dias Ferreira [3], Ranylson Marcello Leal Savedra [4], Melissa Fabíola Siqueira [4], Eduardo Ribeiro de Azevedo [5], Leandro Vinícius Alves Gurgel [1,*]

[1] Group of Physical Organic Chemistry, Department of Chemistry, Institute of Exact and Biological Sciences, Federal University of Ouro Preto, Campus Morro do Cruzeiro, Rua Quatro, 786, Bauxita, 35402-136 Ouro Preto, Minas Gerais, Brazil

[2] Interdisciplinary Research Group on Biotechnology Applied to Agriculture and the Environment, School of Agricultural Engineering, University of Campinas, Avenida Cândido Rondon, 501, 13083-875 Campinas, São Paulo, Brazil

[3] Laboratory of Physical Chemistry and Environmental Chemistry, Department of Chemistry, Institute of Exact and Biological Sciences, Federal University of Ouro Preto, Campus Morro do Cruzeiro, Rua Quatro, 786, Bauxita, 35402-136 Ouro Preto, Minas Gerais, Brazil.

[4] Group of Molecular Simulation of Materials (MolSMat/LabSimCo), Department of Physics, Institute of Exact and Biological Sciences, Federal University of Ouro Preto, Campus Morro do Cruzeiro, Rua Quatro, 786, Bauxita, Ouro Preto 35402-136, Minas Gerais, Brazil

[5] Department of Physics and Interdisciplinary Science, São Carlos Institute of Physics, University of São Paulo, Av. Trabalhador São-carlense, 400, 13566-590, São Carlos, São Paulo, Brazil

[*] Corresponding author. Tel.: +55 31 3559 1707; E-mail address legurgel@ufop.edu.br (L.V.A. Gurgel)

Characterization of the biomaterials

^{13}C solid-state nuclear magnetic resonance (^{13}C SS NMR)

^{13}C SS NMR measurements were carried out in a Bruker Avance 400 spectrometer operating at frequencies of 100.5 MHz (^{13}C) and 400.0 MHz (^{1}H), with a 4-mm (magic angle spinning, MAS) double-resonance probe head operating at 14 kHz. A pneumatic system ensured a spin stability higher than 1 Hz. Typical $\pi/2$ pulse lengths of 4.0 μs (^{13}C) and 3.5 μs (^{1}H) were applied. A proton decoupling field strength of $\gamma B1/2\pi$ = 100 kHz was used. Quantitative ^{13}C SS NMR spectra were measured using the Multi-Cross Polarization (Multi-CP) excitation method described by Johnson and Schmidt-Rohr [87]. Nine cross polarization blocks were employed (1 ms, with an increase in the radio frequency (RF) amplitude of 90-100%). Cross polarization (0.8 ms) before data acquisition using the same increase in the RF amplitude was performed. A recycle delay (2 s) and a repolarization period (0.9 s) were also employed [88].

Fourier Transform infrared (FTIR) spectroscopy

FTIR measurements were carried out on an ABB Bomen spectrometer (Model MB 3000) operating at a resolution of 4 cm^{-1} and 32 scans. The spectrometer was equipped with ZnSe optics and a deuterated triglycine sulfate detector. The spectra of HDSB and HDSB loaded with AO (HDSB-AO) and ST (HDSB-ST) were recorded from 500 to 4000 cm^{-1}. For preparation of KBr pellets (13 mm), 100.0 mg of spectroscopy grade KBr were mixed with 1.0 mg of sample. The solid mixture was pressed in a Pike CrushIR hydraulic press (Model 181-1110, Pike Technologies) at 6 tons (0.5 min).

Energy dispersive X-ray (EDX) spectroscopy

Scanning electron microscopy (SEM) images were recorded on a JEOL scanning electron microscope (Model 6510) coupled to an Oxford EDX spectrometer (Model X-Max) operating at a voltage of 20 keV with a tungsten filament and backscattered electrons detector. The preparation of 13-mm pellets for EDX analyses was made by pressing 100.0 mg of HDSB-AO or HDSB-ST in a Pike CrushIR hydraulic

press (Model 181-1110, Pike Technologies) at 6 tons (0.5 min). The 13-mm pellets (HDSB-AO or HDSB-ST) were sputter-coated with carbon graphite in a JEOL modular high-vacuum coating (Model JEE-420).

Point of zero charge (PZC)

The PZC of HDSB was determined as described by Noh and Schwarz [89]. Into Erlenmeyer flasks (125 mL) containing different amounts of HDSB (0.01, 0.02, 0.1, 0.2, or 0.4 g) was added 20.0 mL of aqueous 0.01 mol L^{-1} $NaNO_3$ solution at an initial pH (pH_i) of 3, 6 or 11 (adjusted with aqueous 0.1 mol L^{-1} NaOH or 0.1 mol L^{-1} HNO_3 solutions). The suspensions were stirred at 130 rpm and 25.0 ± 0.1 ºC for 24 h in a Tecnal orbital shaker incubator (Model TE-424) and the equilibrium pH (pH_e) was measured by a pH meter (Model HI 223, Hanna Instruments). Three curves of pH_e against HDSB weight percentage were obtained and the PZC value was determined at the convergence point of the three curves.

Determination of specific surface area and pore size

The specific surface areas of SB and HDSB were measured on a pore and surface analyzer (Quantachrome, model Nova 1200E) using N_2 adsorption/desorption isotherms at 77.35 K. The samples were degassed at 30 °C for 24 h under a reduced pressure of 0.016 mmHg before measurement. The amounts of N_2 adsorbed and desorbed on SB and HSB were measured over a wide range of pressures (P/P_0 = 2.0 × 10^{-5} - 1.0), where P is the equilibrium pressure and P_0 is the saturation pressure [90]. The specific surface areas were determined by the Brunauer, Emmett, and Teller (BET) method [91], while the pore size distributions were determined by the Barrett, Joyner, and Halenda (BJH) method [92]. The results are shown in **Supplementary Table S1**.

Supplementary Table S1. Textural properties of SB and HDSB

Textural property	SB	HDSB
Specific surface area ($m^2\,g^{-1}$)	14.2	5.4
Average micropore sizes (Å)	40	36
Total pore volumes ($cm^3\,g^{-1}$)	0.015	0.006
Maximum pore diameters (Å)	514	567
Average pore diameters (Å)	21	21

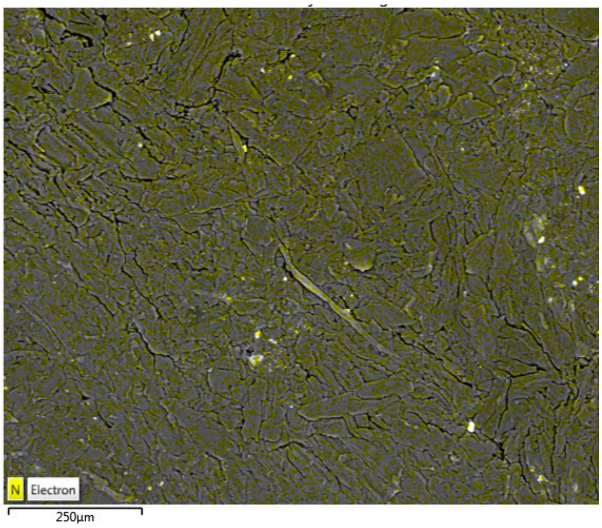

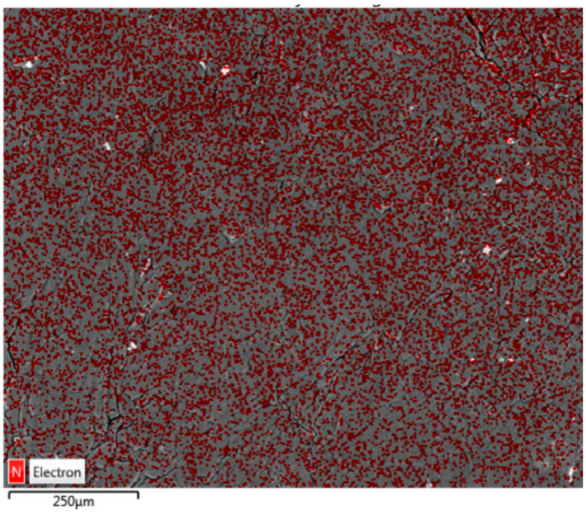

(a) **(b)**

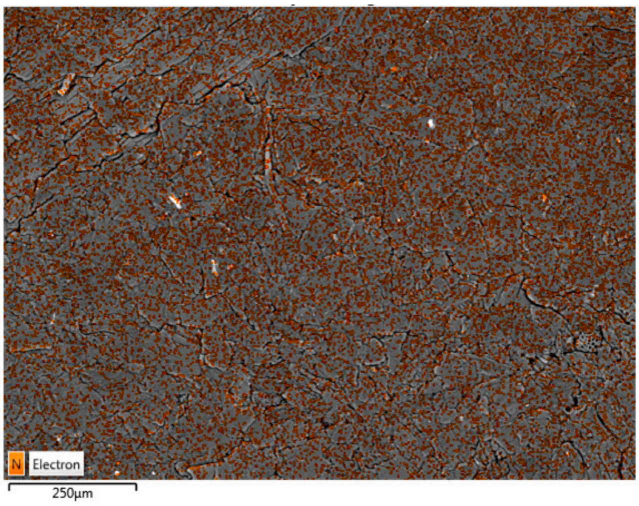

(c)

Supplementary Figure S1. Micrographs of surface mapping of nitrogen of HDSB loaded with (a) AO, (b) ST, and (c) AO and ST, at 100× magnification obtained by MEV-EDX.

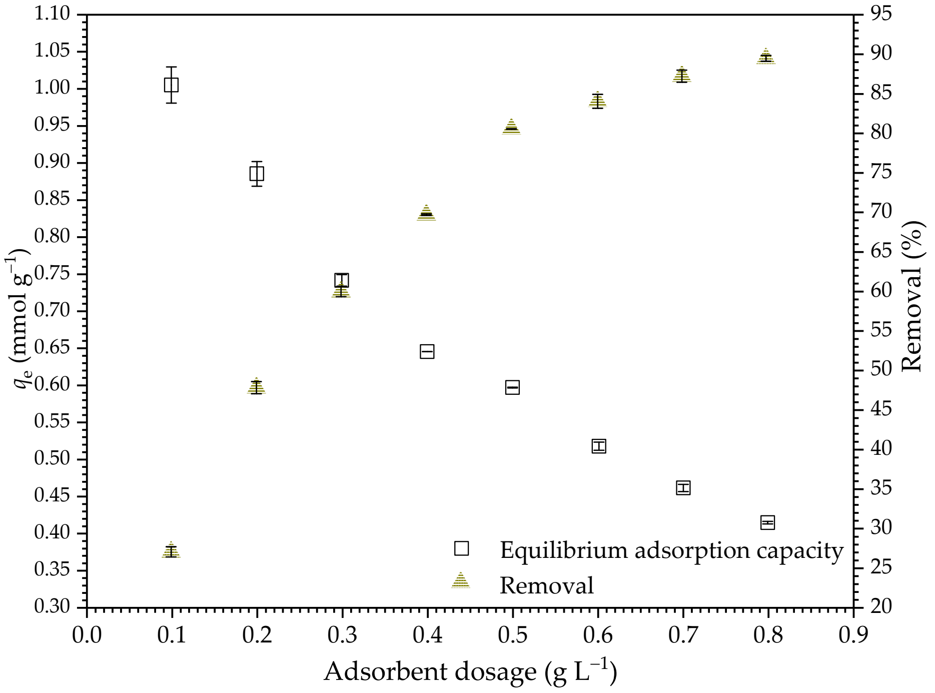

(a)

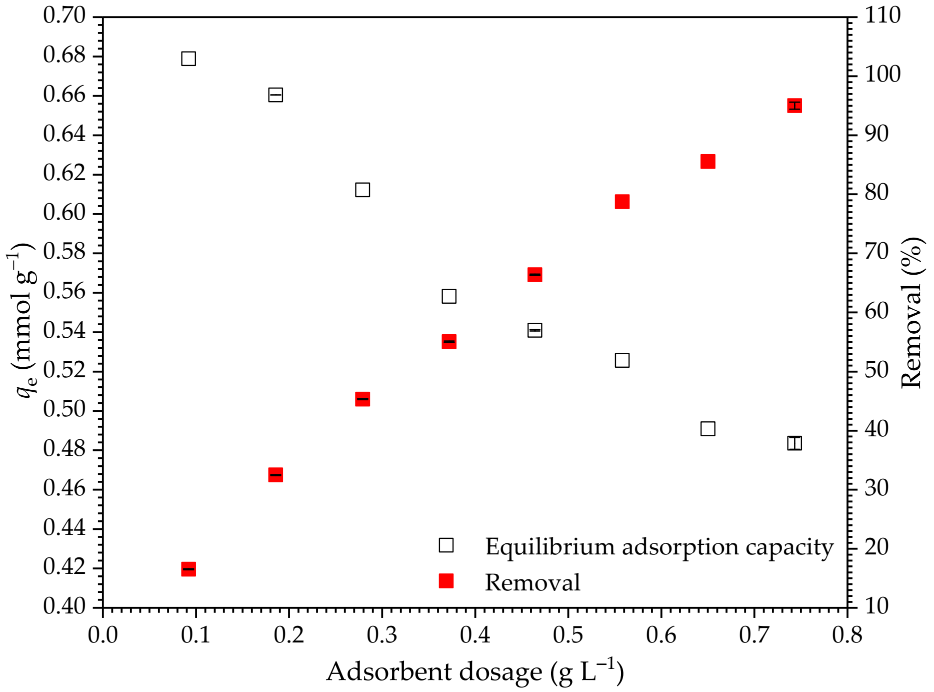

(b)

Supplementary Figure S2. Effect of HDSB dosage on (a) AO and (b) ST adsorption (0.374 mmol L^{-1} AO or ST, 130 rpm, 25.0 ± 0.1 ºC, 24 h, pH 7.0).

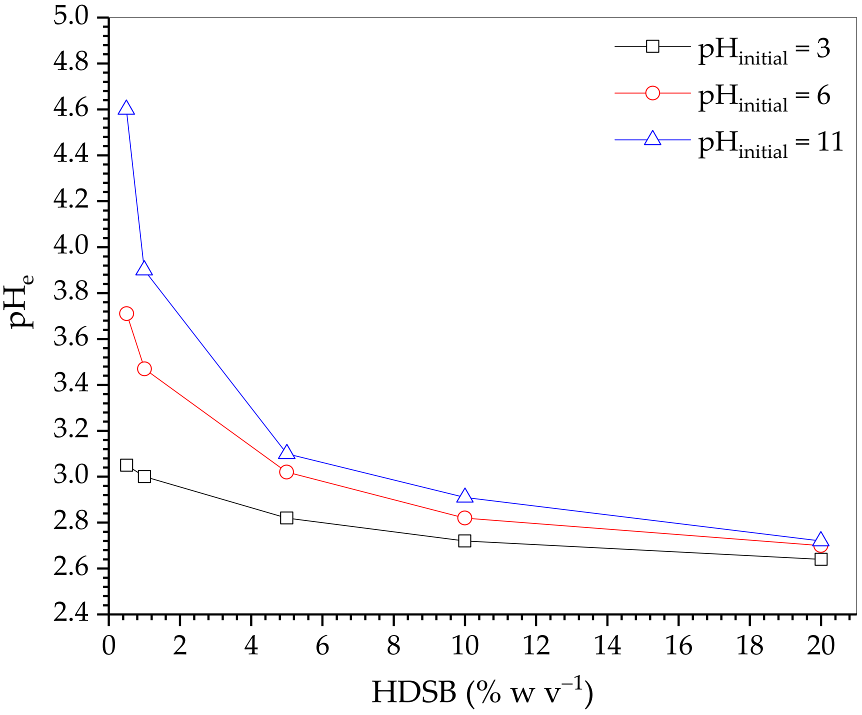

Supplementary Figure S3. Curves of equilibrium pH (pH_e) versus HDSB weight percentage.

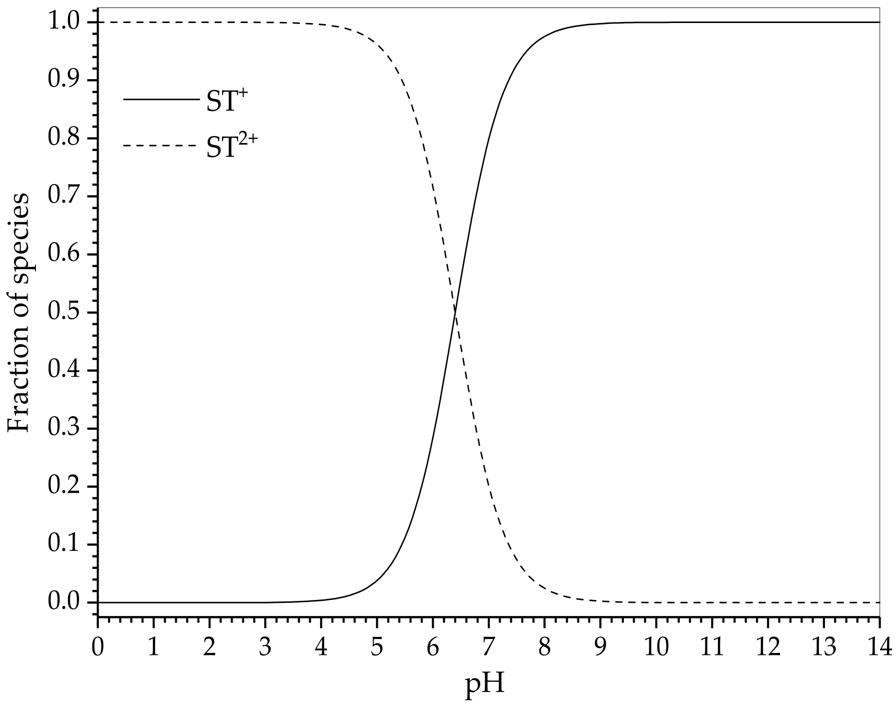

(a)

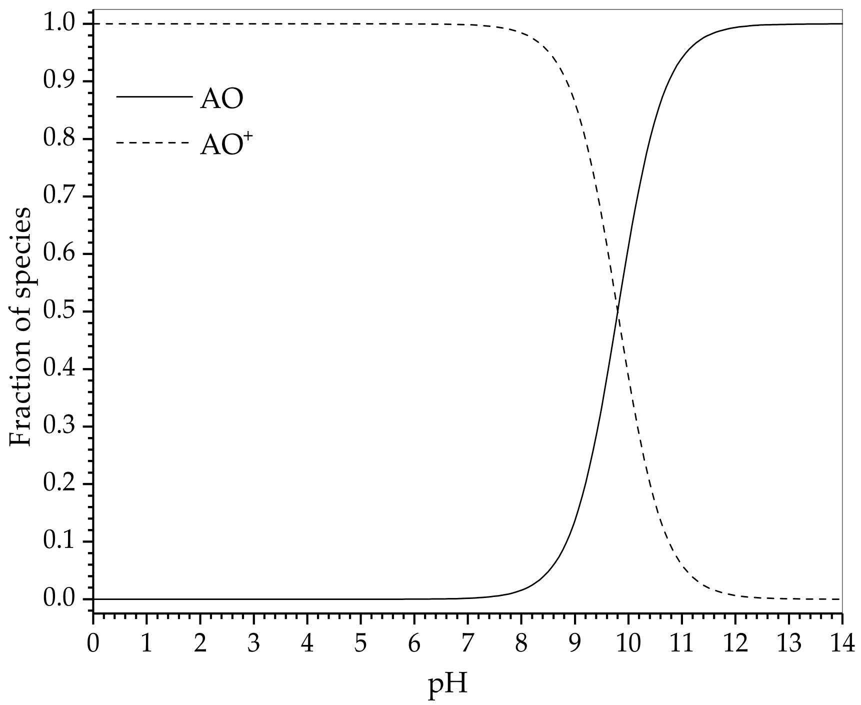

(b)

Supplementary Figure S4. Distribution curves of species of (a) ST and (b) AO, as a function of pH (curves were built using pK_a values of 9.8 and 6.4 for AO and ST [93], respectively).

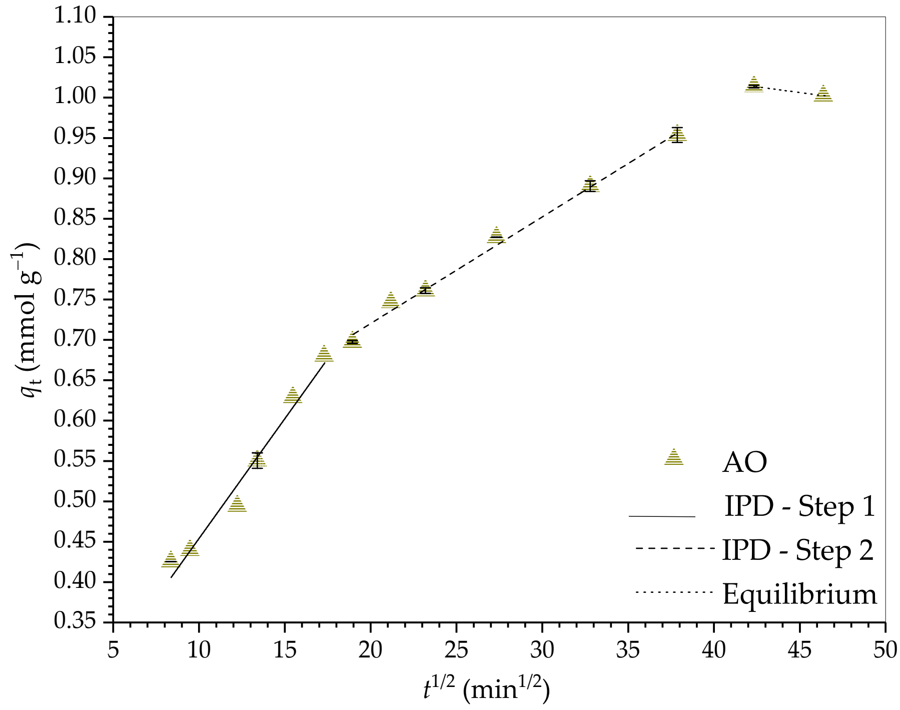

(a)

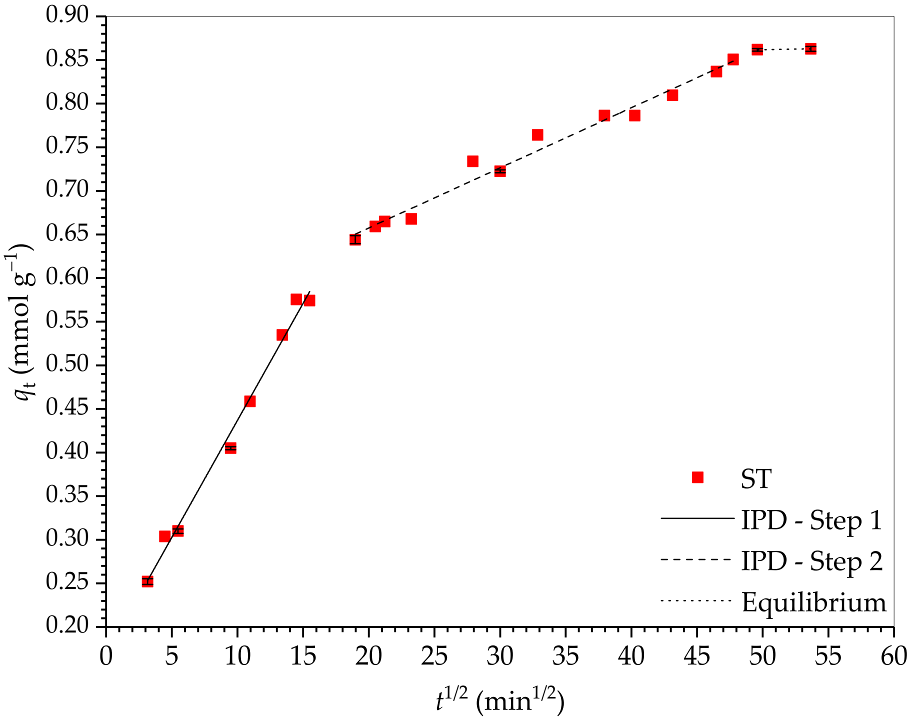

(b)

Supplementary Figure S5. IPD plots for adsorption of (a) AO and (b) ST on HDSB (0.374 mmol L^{-1} AO or ST, 0.2 g L^{-1} HDSB, 130 rpm, 25.0 ± 0.1 ºC, pH 7.0).

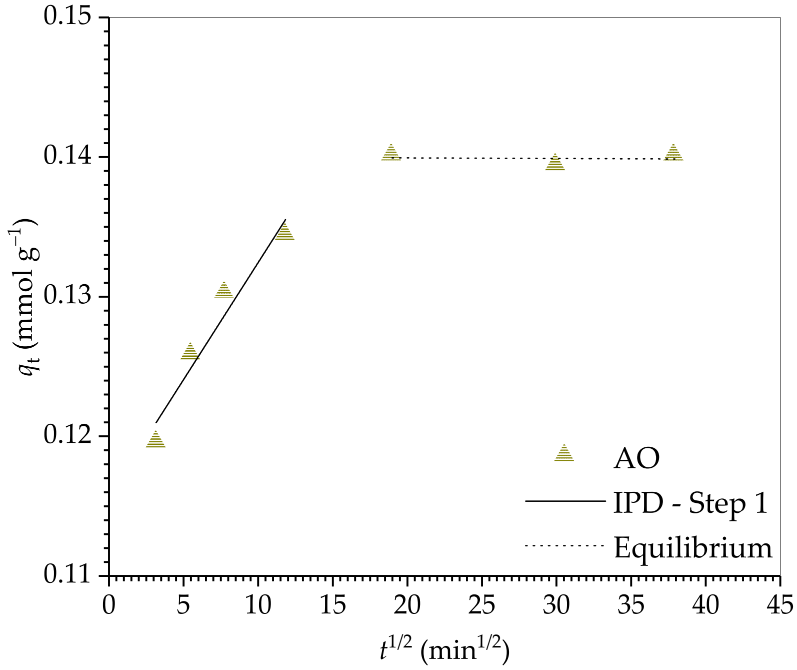

(a)

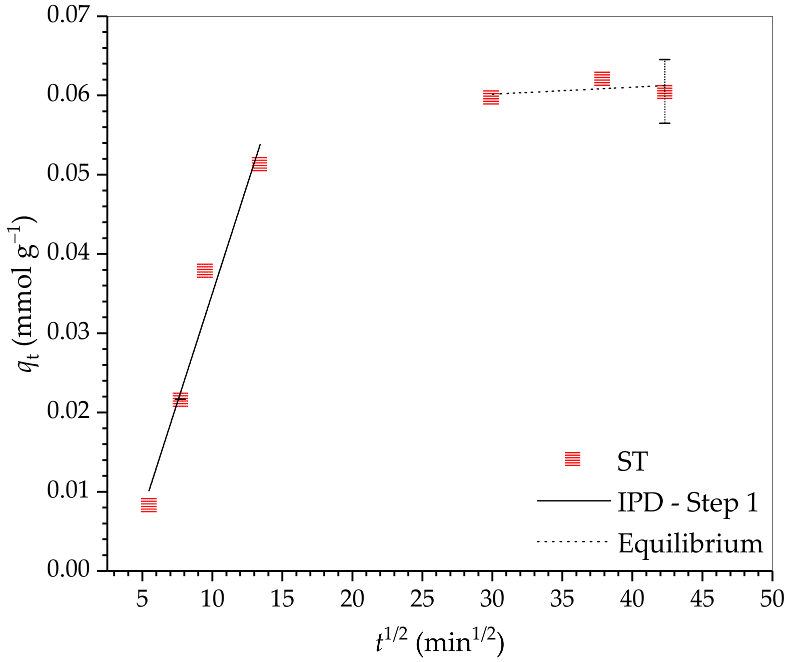

(b)

Supplementary Figure S6. IPD plots for adsorption of (a) AO and (b) ST on SB (0.374 mmol L^{-1} AO and ST, 0.2 g L^{-1} SB, 130 rpm, 25.0 ± 0.1 ºC, pH 7.0).

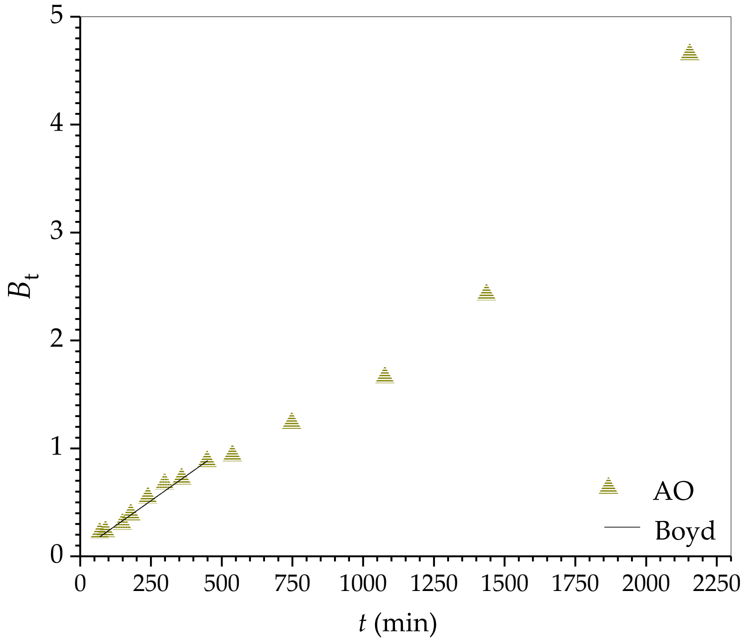

(a)

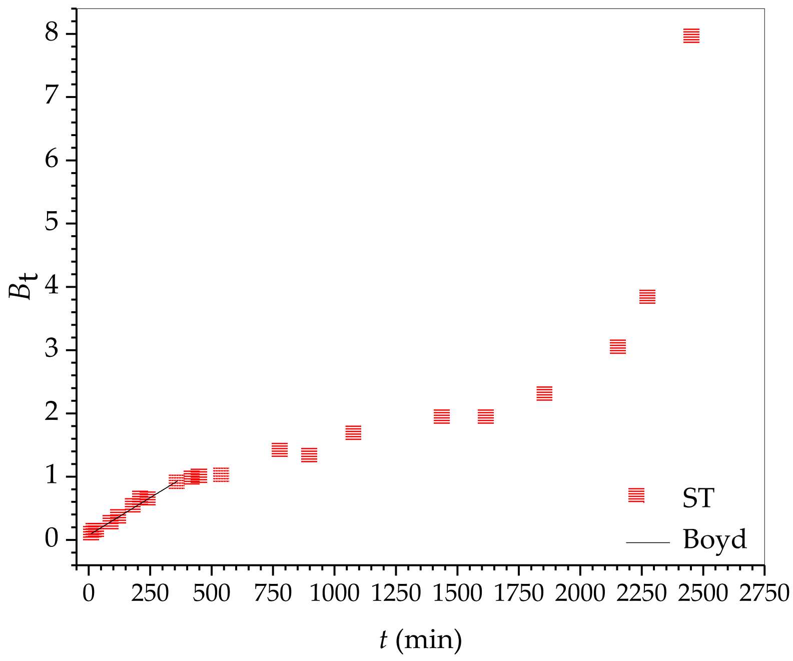

Supplementary Figure S7. Boyd plot for adsorption of (a) AO and (b) ST on HDSB (0.374 mmol L^{-1} AO or ST, 0.2 g L^{-1} HDSB, 130 rpm, 25.0 ± 0.1 ºC, pH 7.0).

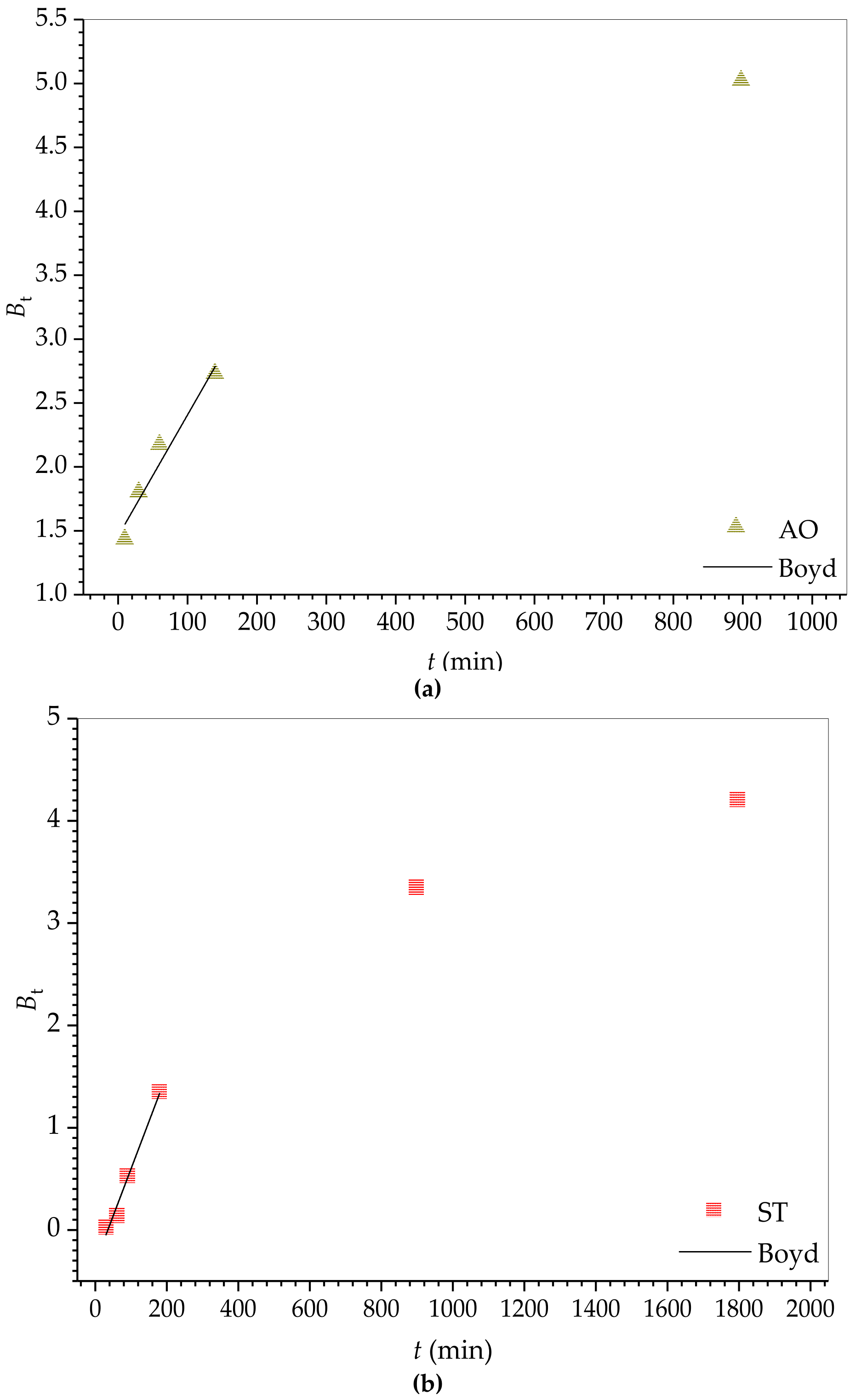

Supplementary Figure S8. Boyd plot for adsorption of (a) AO and (b) ST on SB (0.374 mmol L^{-1} AO or ST, 0.2 g L^{-1} SB, 130 rpm, 25.0 ± 0.1 ºC, pH 7.0).

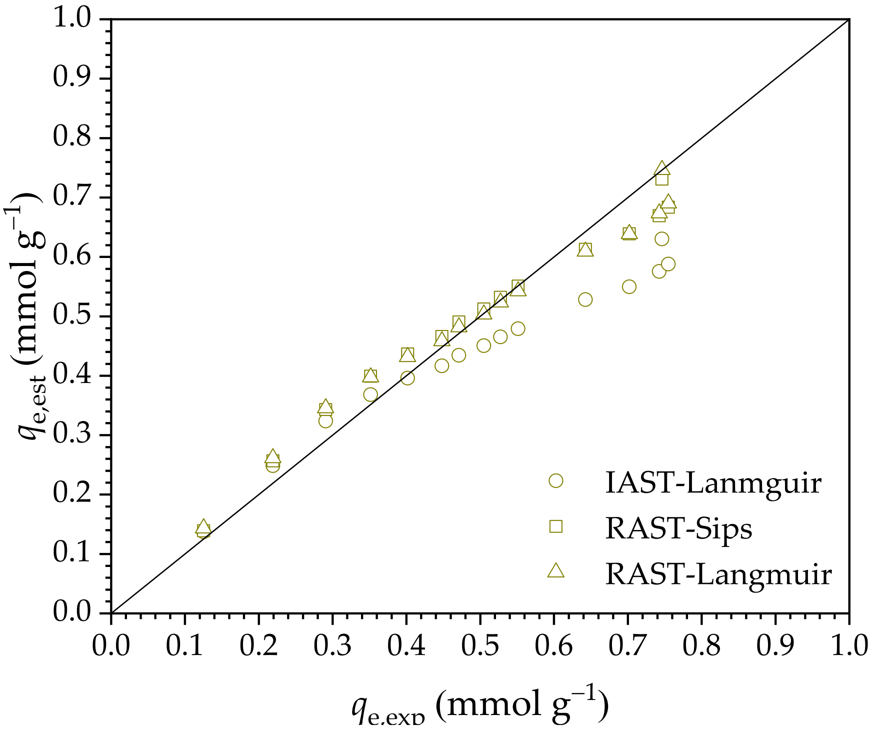

1.0
0.9
0.8
0.7
0.6
0.5
0.4
0.3
0.2
0.1
0.0
$q_{e,est}$ (mmol g^{-1})
IAST-Lanmguir
RAST-Sips
RAST-Langmuir
0.0 0.1 0.2 0.3 0.4 0.5 0.6 0.7 0.8 0.9 1.0
$q_{e,exp}$ (mmol g^{-1})

(a)

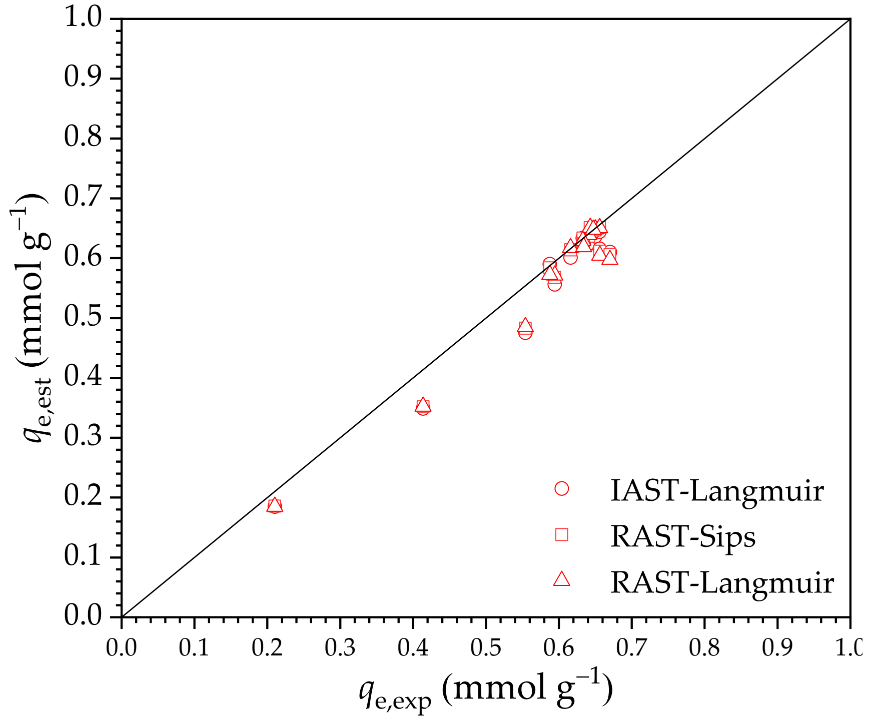

1.0
0.9
0.8
0.7
0.6
0.5
0.4
0.3
0.2
0.1
0.0
$q_{e,est}$ (mmol g^{-1})
IAST-Langmuir
RAST-Sips
RAST-Langmuir
0.0 0.1 0.2 0.3 0.4 0.5 0.6 0.7 0.8 0.9
$q_{e,exp}$ (mmol g^{-1})

(b)

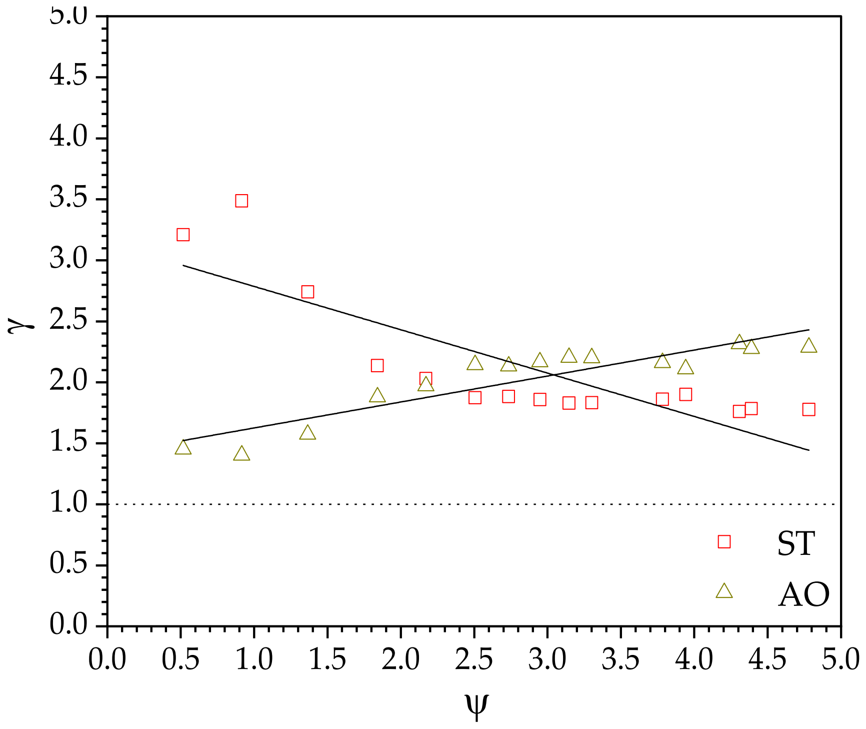

(c)

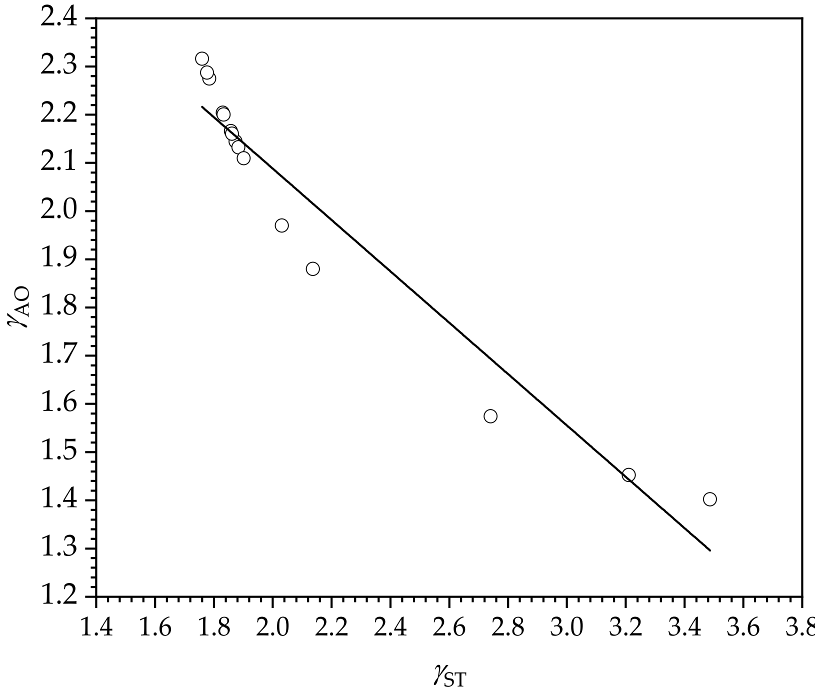

(d)

Supplementary Figure S9. Experimental ($q_{e,exp}$) and estimated ($q_{e,est}$) equilibrium adsorption capacities for (a) AO and (b) ST on HDSB for IAST and RAST models; (c) experimental activity coefficients (γ) for AO and ST, as function of reduced spreading pressure (ψ); and (d) experimental γ_{ST} against experimental γ_{AO} (0.2 g L^{-1} HDSB, pH 7.0, 25.0 ± 0.1 ºC, 130 rpm).

Supplementary Table S2. Mass balance for the desorption and re-adsorption experiments.

<table>
<tr><td rowspan="10">AO</td><td rowspan="2">**Adsorption**</td><td colspan="2">m_{HDSB} / (mg)</td><td>q_e / (mg g^{-1})</td><td>$m_{adsorbed}$ / (mg)</td></tr>
<tr><td colspan="2">20.0</td><td>259.85</td><td>5.20</td></tr>
<tr><td rowspan="4">**Desorption**</td><td colspan="2">$m_{HDSB+AO}$ / (mg)</td><td rowspan="2">$m_{AO,not\ desorbed}$ / (mg)</td><td rowspan="2">$m_{AO,desorbed}$ / (mg)</td></tr>
<tr><td colspan="2">20.0</td></tr>
<tr><td>m_{HDSB} / (mg)</td><td>m_{AO} / (mg)</td><td rowspan="2">2.36</td><td rowspan="2">1.76</td></tr>
<tr><td>15.88</td><td>4.12</td></tr>
<tr><td rowspan="4">**Re-adsorption**</td><td colspan="2">$m_{HDSB+AO}$ / (mg)</td><td rowspan="2">$m_{AO,re\text{-}adsorbed}$ / (mg)</td><td rowspan="2">q_t / (mg g^{-1})</td></tr>
<tr><td colspan="2">20.0</td></tr>
<tr><td>m_{HDSB} / (mg)</td><td>m_{AO} / (mg)</td><td rowspan="2">4.47</td><td>256.75[a]</td></tr>
<tr><td>17.41</td><td>2.59</td><td>405.51[b]</td></tr>
<tr><td rowspan="10">ST</td><td rowspan="2">**Adsorption**</td><td colspan="2">m_{HDSB} / (mg)</td><td>q_e / (mg g^{-1})</td><td>$m_{adsorbed}$ / (mg)</td></tr>
<tr><td colspan="2">20.0</td><td>224.28</td><td>4.48</td></tr>
<tr><td rowspan="4">**Desorption**</td><td colspan="2">$m_{HDSB+ST}$ / (mg)</td><td rowspan="2">$m_{ST,not\ desorbed}$ / (mg)</td><td rowspan="2">$m_{ST,desorbed}$ / (mg)</td></tr>
<tr><td colspan="2">20.0</td></tr>
<tr><td>m_{HDSB} / (mg)</td><td>m_{ST} / (mg)</td><td rowspan="2">1.67</td><td rowspan="2">1.99</td></tr>
<tr><td>16.34</td><td>3.66</td></tr>
<tr><td rowspan="4">**Re-adsorption**</td><td colspan="2">$m_{HDSB+ST}$ / (mg)</td><td rowspan="2">$m_{ST,re\text{-}adsorbed}$ / (mg)</td><td rowspan="2">q_t / (mg g^{-1})</td></tr>
<tr><td colspan="2">20.0</td></tr>
<tr><td>m_{HDSB} / (mg)</td><td>m_{ST} / (mg)</td><td rowspan="2">2.95</td><td>171.51[a]</td></tr>
<tr><td>18.14</td><td>1.85</td><td>334.30[b]</td></tr>
</table>

[a] re-adsorption capacity calculated considering only the amount of re-adsorbed dye in relation to the adsorbent mass contained in $m_{HDSB+dye}$; [b] re-adsorption capacity calculated considering the sum of the re-adsorbed dye mass and the dye mass already present in the bioadsorbent before re-adsorption with respect to the mass of the bioadsorbent contained in $m_{HDSB+dye}$.

Error analysis

$$w_i = \frac{1}{\hat{q}_e} \qquad \text{Supplementary Eq. (S1)}$$

$$\chi^2 = \sum_{i=1}^{N} w_i \, (q_e - \hat{q}_e)^2 \qquad \text{Supplementary Eq. (S2)}$$

where, w_i is the weighting coefficient, q_e is the value obtained experimentally, and $\hat{q}_e$ is the value calculated by the model (PFO, PSO or Elovich).

$$\chi^2_{red} = \frac{\chi^2}{\upsilon} \qquad \text{Supplementary Eq. (S3)}$$

where, υ is the number of degrees of freedom ($\upsilon = N - P$), i.e., the difference between the number of experimental data points (N) and the number of variables (P) of the model (PFO or PSO).

$$R^2 = 1 - \frac{\sum(\hat{q}_e - q_e)^2}{\sum(\hat{q}_e - \overline{q_e})^2} \qquad \text{Supplementary Eq. (S4)}$$

where $\overline{q_e}$ (mg g^{-1}) is the average of the values of q_e.

$$R^2_{adj} = 1 - \frac{(1 - R^2)(N-1)}{(N-P-1)} \qquad \text{Supplementary Eq. (S5)}$$

The SSR (Sum Squared Residues) was calculated by Eq. (S6):

$$SSR = \sum_{i=1}^{N} (\hat{q}_e - q_e)^2 \qquad \text{Supplementary Eq. (S6)}$$

where, q_e is experimental value and $\hat{q}_e$ is the calculated value by the model.

References

87. Johnson, R. L.; Schmidt-Rohr, K., Quantitative solid-state 13C NMR with signal enhancement by multiple cross polarization. *J. Magn. Reson.* **2014,** 239, 44-49. http://dx.doi.org/10.1016/j.jmr.2013.11.009

88. Bernardinelli, O. D.; Lima, M. A.; Rezende, C. A.; Polikarpov, I.; deAzevedo, E. R., Quantitative 13C MultiCP solid-state NMR as a tool for evaluation of cellulose crystallinity index measured directly inside sugarcane biomass. *Biotechnol. Biofuels* **2015,** 8, (1), https://doi.org/110.10.1186/s13068-015-0292-1

89. Noh, J. S.; Schwarz, J. A., Effect of HNO_3 treatment on the surface-acidity of activated carbons. *Carbon* **1990,** 28, (5), 675-682. https://doi.org/10.1016/0008-6223(90)90069-B

90. Guo, J.; Catchmark, J. M., Surface area and porosity of acid hydrolyzed cellulose nanowhiskers and cellulose produced by Gluconacetobacter xylinus. *Carbohydr. Polym.* **2012**, 87, 1026-1037. https://doi.org/10.1016/j.carbpol.2011.07.060

91 Brunauer, S.; Emmett, P.H.; Teller, E., Adsorption of gases in multimolecular layers. *J. Am. Chem. Soc.* **1938**, 60, 309–319, https://doi.org/10.1021/ja01269a023

92 Barrett, E.P.; Joyner, L.G.; Halenda, P.P., The determination of pore volume and area distributions in porous substances. I. Computations from nitrogen isotherms. *J. Am. Chem. Soc.* **1951**, 73, 373–380. https://doi.org/10.1021/ja01145a126

93. Sabnis, R. W., *Handbook of Biological Dyes and Stains: Synthesis and Industrial Applications*. John Wiley & Sons: 2010; p 521. https://doi.org/10.1002/9780470586242.ch1
